# Engineered conductive pili enable high-efficiency photosynthetic electron extraction in biophotovoltaics

**DOI:** 10.1038/s41467-026-72407-7

**Published:** 2026-04-25

**Authors:** Haowei Wang, Yanping Zhang, Yin Li, Huawei Zhu

**Affiliations:** 1 State Key Laboratory of Microbial Diversity and Innovative Utilization, Institute of Microbiology, Chinese Academy of Sciences, Beijing, China; 2 University of Chinese Academy of Sciences, Beijing, China

**Keywords:** Biotechnology, Applied microbiology, Synthetic biology, Renewable energy

## Abstract

The efficient extraction of electrons from photosynthetic microorganisms remains a critical challenge in living biophotovoltaics (BPV). While nanomaterials can facilitate electron transport, their stochastic adsorption leads to inefficient material-wasteful interfaces. Here, we demonstrate a controllable approach to direct the targeted assembly of gold nanoparticles (AuNPs) onto the type IV pili of *Synechocystis* sp. PCC 6803 by using a genetically encoded gold-binding peptide. This approach creates a spatially precise conductive nano-bio interface on the cell envelope that serves as a dedicated electron conduit between photosynthetic electron transport chains (PETCs) and electrodes. This nano-bio interface enhances electron transfer through synergistic improvements in interfacial charge transfer and biofilm density, ultimately yielding a four-fold increase in photocurrent density, while using two orders of magnitude less gold than non-targeted strategies. Moreover, the AuNPs can be transferred from inactivated to fresh cells, indicating a potential pathway for long-term stability. This work establishes a generalizable strategy for the rational design of conductive interfaces on living cells, with implications for biophotovoltaics, microbial electrosynthesis, and next-generation biohybrid devices.

## Abstract (web summary)

Inefficient extracellular electron transfer of cyanobacteria impedes power output in biophotovoltaics. Here, the authors assemble a gold conductive layer on the cell surface by attaching gold-binding peptide to the pili structure, achieving a four-fold increase in photocurrent generation.

## Introduction

Oxygenic photosynthetic microorganisms, including eukaryotic algae and cyanobacteria, absorb light and oxidize water to generate oxygen and high energy electrons^1^. A small portion of excess electrons can be exported across the cell envelope and released into external environments, a process known as exoelectrogenesis or extracellular electron transfer (EET)^2,3^. Depending on this process, a cutting-edge technology known as biophotovoltaics (BPV) have been developed for solar-to-electricity conversion^4,5^. BPV is a bioelectrochemical system where photosynthetic microorganisms are typically interfaced with an anode and the released electrons are flow through an external circuit to the cathode, thereby generating a photocurrent^6^. It was estimated that the achievable current densities of BPV range from 340 to 2460 μA cm^−2^, dependent on light photon flux in different geographical locations^7^. However, the reported electrical current outputs are generally lower than this value by two or three orders of magnitude, and the lab-built BPVs were only able to run items with power demand lower than 1 mW^8^. This was ascribed to the weak exoelectrogenesis activity of photosynthetic microorganisms, thus hindering the electron transport from intracellular photosynthetic electron transport chains (PETCs) to extracellular electrode^9^.

Many efforts have been made to improve the electron transfer process between the PETCs and the electrode^10^. Most studies highlighted the importance of electrode materials and structures. Electrode engineering generally improves the electrical conductivity and increases the electroactive surface area, thereby augmenting the ability for electron collection^11^. We previously constructed two generations of microbial consortia-based BPV systems by directing photosynthetic energy flow toward specialized electroactive bacteria, resulting in great improvement in power output and system lifetime^12,13^. However, this strategy was limited by theoretical maximum energy efficiency due to the involvement of the carbon fixation process. In addition, the cell membrane and cell wall have been identified as the primary barriers for photosynthetic electron extraction^14^. To this end, some bioengineering strategies have been proposed to eliminate electron transfer barriers or create electron transfer pathways. For instance, removing of structural barriers such as the outer membrane or exopolysaccharide matrix by gene deletion exhibited a remarkable improvement in photocurrent^15,16^. The heterogeneous expression of electron transfer proteins, such as OmcS and MtrA from specialized exoelectrogens, into cyanobacteria resulted in photocurrent improvement by ninefold and twofold, respectively^17,18^. A further substantial improvement in solar-powered electricity generation relies on a complete biological electron conduit. However, it was difficult in the active assembly of a full protein complex in the cell membrane of photosynthetic microorganisms.

Nanomaterials, such as metal nanomaterials and carbon nanomaterials, possess unique properties including tunable surface properties, larger specific surface area, and excellent electrical properties^19–21^. These nanomaterials can function as an abiotic electron transfer channel to aid the transmembrane electron transfer in photosynthetic microorganisms^22^. For instance, the single-walled carbon nanotubes located in the peripheral regions of the *Synechocystis* cells could serve as an auxiliary electron conduit^23^. This led to a significant decrease in surface impedance and an approximately 15-fold increase in photocurrent. In addition, polydopamine (PDA) encapsulation of *Synechocystis* cells through self-polymerization of dopamine results in enhancement in cell-electrode adhesion and charge extraction^24^. However, the fabrication of photosynthetic-nanomaterial hybrids largely relied on physical or chemical adsorption between nanomaterials and cells^25^. These stochastic interactions would result in a random, uncontrolled, and material-inefficient distribution of conductive nanomaterials on the cell surface or inside the cells, thereby limiting their actual effects in assisting electron transfer^26^. The lack of spatial precision prevents the formation of optimal electron conduits and wastes the cell’s innate capacity to guide functional assembly. A more elegant solution lies in creating a direct, abiotic conductive pathway that seamlessly integrates with the cellular architecture.

**Fig. 1:**
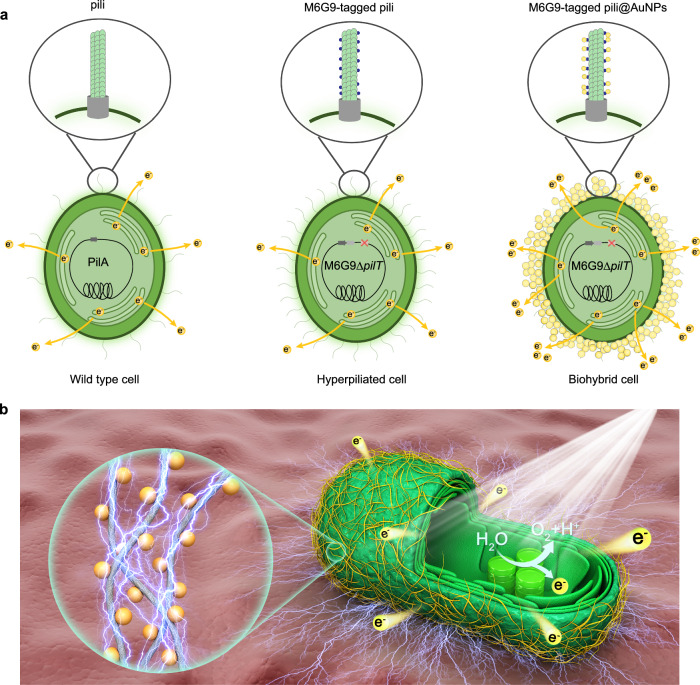
Engineering electrically conductive pili in *Synechocystis* enhance export of photosynthetic electrons. **a** The wild type of *Synechocystis* naturally produces type IV pili (left). An engineered hyperpiliated *Synechocystis* was generated by deletion of *pilT* encoding retraction ATPase and genetically fusion of type IV pili with a gold binding peptide M6G9 (middle). The M6G9-tagged pili triggered the targeted assembly of AuNPs via the specific interaction between M6G9 and AuNPs (right). **b** Schematic representation of electrically conductive layer enhancing solar-powered electricity generation. Targeted binding of AuNPs on M6G9-tagged pili enables a cohesive electron-conducting network on cell surface, forming a conductive nano-bio interface. The intracellular electrons derived from photocatalytic water-splitting by photosystems are transferred into the outside by this conductive interface for electricity generation.

In this work, we aimed to engineer the cell to actively direct nanomaterials to specific locations so as to achieve a controllable assembly of conductive layer on the cell envelope of cyanobacteria (Fig. 1). Solid-binding peptides are ideal molecular tools for this purpose, as they can be genetically encoded to selectively bind to inorganic materials, with high affinity and specificity^27^. We first identified a high-affinity gold-binding peptide (GBP) and genetically fused it to the major subunit of type IV pilus of *Synechocystis* sp. PCC 6803 (hereafter referred to as *Synechocystis*). This engineered *Synechocystis* served as a template for the controlled and precise localization of gold nanoparticles (AuNPs), forming conductive “nanowires” that directly wire the intracellular PETC to an external electrode. This conductive nano-bio interface decreased electron transfer resistance, dramatically enhanced photocurrent generation, and achieved this with a substantially reduced consumption of AuNPs. This work establishes a generalizable paradigm for the rational design of hybrid electronic interfaces on living cells, with implications that extend beyond energy harvesting to microbial electrosynthesis and engineered living materials.

## Results

### Identification of a high affinity gold-binding peptide

To establish a programmable interface for electron extraction, we first sought a molecular tool for precise nanomaterial assembly. We selected gold nanoparticles (AuNPs) as the conductive abiotic component due to their excellent conductivity and favorable biocompatibility demonstrated in bioinorganic hybrid systems^28,29^. Our strategy was to use GBP to mediate the specific recognition and specific attachment of AuNPs to the surface of *Synechocystis* cells (Fig. 1). To identify a GBP with high affinity to AuNPs, we firstly determined the binding capacity of a panel of GBP candidates displayed on the cell surface of *Escherichia coli*^30^.

**Fig. 2:**
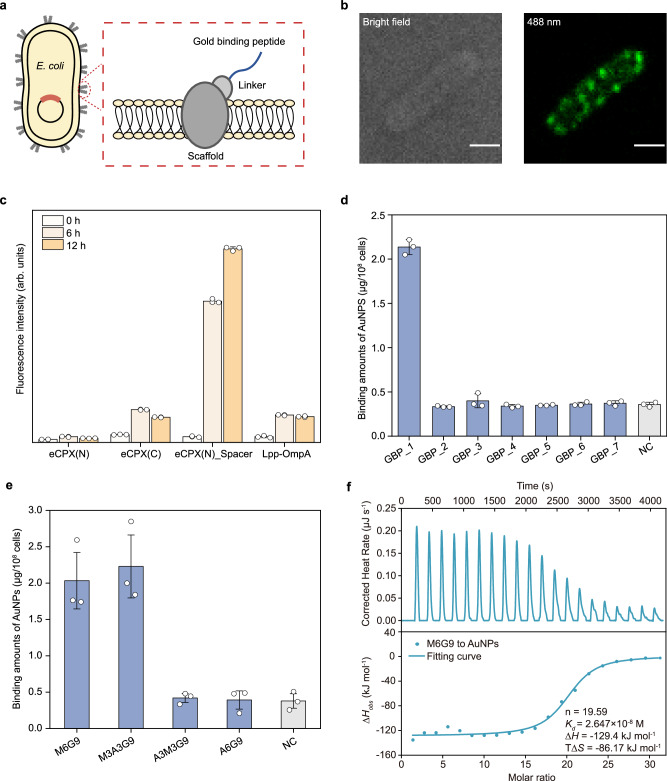
Screening of GBPs with high affinity to AuNPs using *E. coli* surface display system. **a** The diagram of an engineered *E. coli* surface display system. An outer membrane protein was used as scaffold for the fusion expression of targeted peptides or proteins. **b** Confocal microscopy images show green fluorescence was distributed near the cell envelope of *E. coli* cell when eGFP was displayed using eCPX(N)_Spacer system. *E*_*x*_/*E*_*m*_ = 488 nm/510-530 nm. Scale bar: 1 μm. **c** Surface display capacity of different surface display systems in *E. coli* using eGFP as reporter. The scaffold protein eCPX was used for eGFP fusion at either N-terminal or C-terminal or addition of a spacer, resulting in three display systems. Data are presented as mean values ± SD from *n* = 3 independent experiments. **d** The binding amount of AuNPs by different GBPs displayed on *E. coli* surface using eCPX(N)_Spacer system. NC: negative control. Data are presented as mean values ± SD from *n* = 3 independent experiments replicates. **e** The binding amount of AuNPs by M6G9 mutants displayed on *E. coli* surface using eCPX(N)_Spacer system. NC: negative control. Data are presented as mean values ± SD from *n* = 3 independent experiments. **f** ITC analysis of M6G9-AuNPs interaction. The upper panel shows the raw heat flow curves over time and the lower panel shows the fitting curves of Δ*H* versus the molar ratio (M6G9/AuNPs). Source data are provided as a Source Data file.

The surface display system of *E. coli* was developed by fusion expression of targeted peptide or protein with an outer membrane scaffold protein (Fig. 2a). Two reported scaffold proteins, including the enhanced circularly permutated outer membrane protein X (eCPX) and the chimaera of lipoprotein and outer membrane protein A (LPP-OmpA) were used separately. Fusion expression of an enhanced green fluorescent protein (eGFP) in N-terminal or C-terminal of these two scaffolds was used to evaluate its surface display capacity. The results showed that the surface display system of eCPX(N)_Spacer that displaying eGFP on the N-terminal of eCPX with a spacer (GTSGQ) between the signal peptide and eGFP, exhibited the highest fluorescence intensity, once the fusion expression cassette was induced with isopropyl-β-d-thiogalactoside (IPTG) for 6 h or 12 h (Fig. 2c). By contrast, the display efficiencies of surface display systems eCPX(C) and LPP-OmpA were significantly lower than eCPX(N)_Spacer, although both systems are working. The sharp fluorescence difference of eCPX(N) and eCPX(N)_Spacer indicated the critical role of the spacer sequence in ensuring proper signal peptide cleavage and preventing interference with the conformation of the target peptide or protein. Confocal laser scanning microscopy (CLSM) revealed that the green fluorescence of *E. coli* cells using eCPX(N)_Spacer system preferentially distributed near to the cell envelope (Fig. 2b). To confirm the surface exposure of eGFP, protease accessibility assay and antibody labeling were employed. Fluorescence in *E. coli* cells with surface-displayed eGFP significantly decreased after proteinase treatment for 1 h, while cytosolic-expressed eGFP was unaffected (Supplementary Fig. 1a). In addition, flow cytometry analysis revealed that only induced *E. coli* cells with surface eGFP expression was successfully labeled by an anti-eGFP antibody modified with far-red fluorescence (Supplementary Fig. 1b). These orthogonal approaches confirm the surface-localized display of fusion proteins, indicating the surface display system of eCPX(N)_Spacer was well-worked in *E. coli*.

Using this optimized system, seven reported GBPs (peptide sequences listed in Supplementary Table 1) were displayed on *E. coli* surface. After the inducible expression of the fused GBPs, *E. coli* cells and AuNPs solutions were mixed for 3 h to trigger the assembly of AuNPs on *E. coli* surface. The amount of binding AuNPs was measured using inductively coupled plasma mass spectrometry (ICP-MS). A gold binding peptide GBP_1, also called M6G9, which consists of six methionine and nine glycine residues (MMMGGGMGGGMGGGM), showed great affinity to AuNPs with a binding amount of 2.13 ± 0.07 μg AuNPs per 10^8^ cells, while the others showed negligible affinity (Fig. 2d). To identify critical binding motifs and residues, three M6G9 mutants, including M3A3G9, A3M3G9 and A6G9 (Supplementary Table 1), were generated and their binding capacity to AuNPs were evaluated using *E. coli* surface display system. It was shown that the mutant M3A3G9 retained binding capacity, while mutants A3M3G9 and A6G9 lost it completely (Fig. 2e). This indicated the methionine residues in N-terminal of M6G9, rather than those in GGGM repeat units, critically contributed to the binding affinity with AuNPs. X-ray photoelectron spectroscopy (XPS) analysis revealed that in the Au 4*f* spectrum, AuNPs show two peaks at binding energies of 83.7 and 87.3 eV (Supplementary Fig. 2a), corresponding to the spin-orbit splitting of Au 4*f*_7/2_ and Au 4*f*_5/2_, respectively, which align well with the values of Au^0^. Upon interaction with M6G9, two peaks with a little higher binding energy appeared, indicating the presence of the oxidation state of Au^1+^. In the S 2*p* spectrum of M6G9, there are two peaks with binding energy at 163.0 and 164.2 eV, corresponding to the spin-orbit splitting of S 2*p*_3/2_ and S 2*p*_1/2_, respectively (Supplementary Fig. 2b). Upon binding with AuNPs, two distinct peaks with lower binding energy appeared in the S 2*p* spectrum of M6G9. These results suggested that AuNPs covalently bind to M6G9 through Au-S bonding. Isothermal titration calorimetry (ITC) measurement further confirmed the high binding capacity of M6G9, revealing a binding affinity of 26.5 nM and a stoichiometry of about 20 (Fig. 2f). By contrast, ITC analysis showed no affinity of A6G9 to AuNPs (Supplementary Fig. 3). Therefore, M6G9 peptide was chosen for further experiments.

### Programming *Synechocystis* pilus to display M6G9

**Fig. 3:**
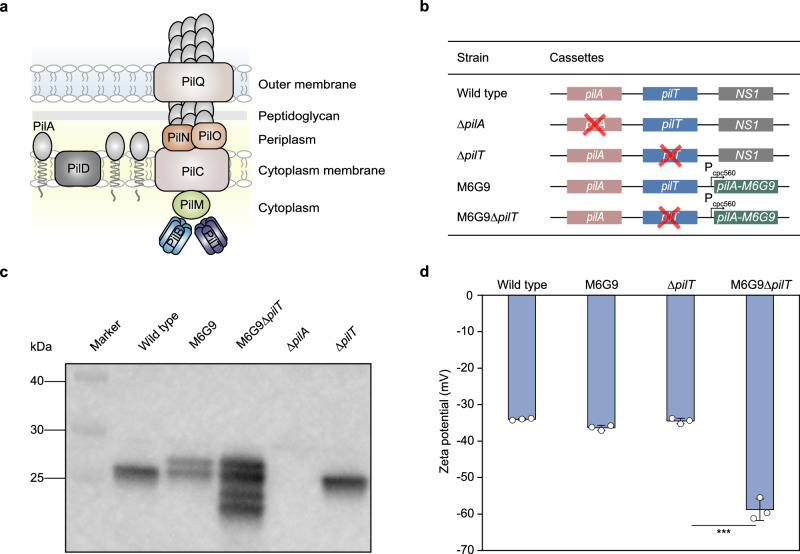
Construction of M6G9-tagged pili in *Synechocystis.* **a** The schematic of type IV pili architecture in *Synechocystis*. **b** The engineered *Synechocystis* strains with different genotypes. The horizontal boxes represent the genes. The red crosses indicate the genes were knocked out. **c** Immunoblotting analysis of whole-cell extracts of different strains. The protein bands were probed using an anti-PilA polyclonal antibody. **d** The zeta potentials of different strains. Two-sided *t*-test was used to determine the significance (^***^p < 0.001). The *p* value in **d** is 0.00016. Data are presented as mean values ± SD from *n* = 3 independent experiments. Source data are provided as a Source Data file.

Choosing a structural protein to anchor M6G9 is crucial for constructing a conductive layer on the cell envelope. The cell envelope of *Synechocystis* consists of a periplasmic space with a peptidoglycan layer, an outer membrane, a surface layer (S-layer) and extracellular appendages such as type IV pili (Fig. 3a). PilA1 is a major pilin protein, which constitutes the majority of type IV pili structure^31^. Due to the polymeric nature of the pilus, fusion expression with PilA1 could significantly enhance surface display levels in *Synechocystis*. Furthermore, it was reported that *Synechocystis* pilus could be genetically engineered for surface display of affibody proteins^32^, providing a theoretical framework to explore the potential of the pilus structure to facilitate the controlled enrichment of AuNPs.

Firstly, a thick pilus-deficient mutant (Δ*pilA*) and a hyperpiliated mutant (Δ*pilT*) were constructed to investigate whether the pilus structure contributes to the exoelectrogenesis in *Synechocystis*. The phenotypes of two mutants and wild-type were confirmed through negative stain transmission electron microscopy (TEM) (Supplementary Fig. 4). Notably, the photocurrent densities generated by both pilus mutants were similar to that of wild-type (Supplementary Fig. 5), which was consistent with previous studies^33,34^. Therefore, the pilus did not contribute to photocurrent generation, enabling it as an ideal anchor structure for M6G9. Subsequently, the M6G9 fusion was positioned at the C-terminus of PilA1, flanked by a flexible linker (GSSSGSS) for accessibility. Since the N-terminus of PilA1 forms a hydrophobic helix embedded within the pilus rod^35^, rendering it unsuitable for peptide fusion (Supplementary Fig. 6). The fusion expression cassette containing PilA1 and M6G9 coding sequences was integrated into the genome of wild-type *Synechocystis* through homologous recombination, which was verified by colony PCR and DNA sequencing (Supplementary Fig. 7).

Functional expression of the PilA-M6G9 fusion cassette in *Synechocystis* was verified by immunoblotting against PilA1 affibody. We initially chose the *pilA1* site as the integration site to replace native *pilA1* with the PilA-M6G9 fusion cassette. However, the expected band was not observed in the immunoblotting analysis (Supplementary Fig. 8a), which might ascribed to the lack of C-terminal lysine trimethylation, a post-translational modification essential for maintaining the functional pilus structure^36^. Therefore, native PilA1 is necessary to the assembly and functionality of the pilus. To retain the expression of native *pilA1*, PilA-M6G9 fusion cassette was integrated into a neutral site I (NSI), resulting in strain M6G9 (Fig. 3b). The expression level of the fusion cassette was optimized using two promoters P_rbcL_ and P_cpc560_. Immunoblotting analysis showed an additional larger protein band for fusion mutant compared with that of wild-type, which was attributed to the expression of PilA-M6G9 (Fig. 3c). Notably, the stronger promoter P_cpc560_ resulted in a higher expression level, whereas no detectable expression was observed with P_rbcL_ (Supplementary Fig. 8b). In addition, the larger-than-expected bands (19.4 kDa for PilA-M6G9 and 17.6 kDa for PilA1) observed in the blot indicated that both native PilA1 and PilA-M6G9 fusion were undergone post-translational modifications, although it was not known whether the modifications were the same for both.

To further enhance the expression level of PilA-M6G9, gene *pilT* was deleted in strain M6G9 to obtain the strain M6G9Δ*pilT* (Fig. 3b). This hyperpiliated mutant was expected to increase the amount of surface-exposed M6G9. Immunoblotting analysis revealed that the knockout of *pilT* led to increased expression level for both the PilA-M6G9 fusion and native PilA1 (Fig. 3c). The presence of two smaller bands in the strain M6G9Δ*pilT* suggested that some proteins might not undergo post-translational modifications^37^. Meanwhile, TEM analysis showed that the strain M6G9Δ*pilT* exhibited a functional pili structure similar to the strain Δ*pilT* (Supplementary Fig. 4). It was found that the strain M6G9Δ*pilT* in the solution exhibited a phenotype of suspension without sedimentation, compared to the strain Δ*pilT* (Supplementary Fig. 9). This could be attributed to a more negative value of zeta potential (Fig. 3d), thus leading to stronger electrostatic repulsion between the cells. These results demonstrated that the GBP of M6G9 was successfully displayed on the pili structure of *Synechocystis*. The engineered strain M6G9Δ*pilT* was used in the subsequent construction of photosynthetic-nanomaterial hybrids.

### Hybrid pilus facilitated targeted enrichment of AuNPs

**Fig. 4:**
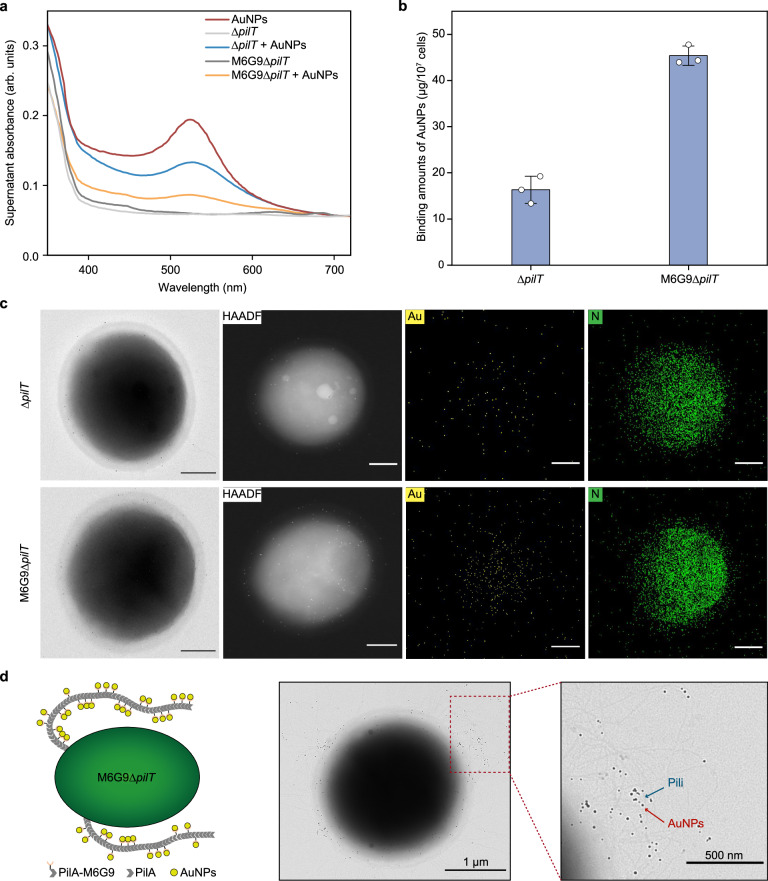
Targeted enrichment of AuNPs mediated by M6G9-tagged pili. **a** The absorption spectra of the supernatants of *Synechocystis* cells incubated with or without AuNPs. The red line represents AuNPs solution without *Synechocystis* cells. **b** The binding amount to AuNPs of two engineered *Synechocystis* strains. Data are presented as mean values ± SD from *n* = 3 independent experiments. **c** The STEM images and EDX mapping of *Synechocystis*-AuNPs hybrids. The yellow represents Au atoms while green represents N atoms. Scale bar: 0.5 μm. **d** Schematic of targeted enrichment of AuNPs on the pili of *Synechocystis* (left) and the corresponding TEM images (right). The experiments in **c**, **d** were repeated three times independently with similar results. Source data are provided as a Source Data file.

The enrichment of AuNPs on the pilus was performed by incubating fresh cells with AuNPs at a concentration of 25 μg mL^−1^ in BG11 medium for 2 h. To evaluate the binding capacity of M6G9Δ*pilT*, the UV-visible absorption spectra of different supernatants following the enrichment process were compared. As shown in Fig. 4a, the aqueous solution of AuNPs used in this study exhibited an characteristic absorption peak near 530 nm, which was attributed to the plasmonic band of AuNPs^38^. After incubation with *Synechocystis* cells, whatever for strain M6G9Δ*pilT* or strain Δ*pilT*, a decrease in this characteristic peak was observed, indicating AuNPs were adsorbed by *Synechocystis*. It was reported that the extracellular polymeric substances could naturally bind AuNPs^39^, which might contribute to the absorption decrease in the supernatant of strain Δ*pilT*. As for strain M6G9Δ*pilT*, the characteristic peak at 530 nm almost disappeared completely, showing a much higher binding capacity than strain Δ*pilT*. This difference was attributed to the enhanced binding capacity of hybrid pilus composed of M6G9 binding peptide. The binding of AuNPs by *Synechocystis* cells was further confirmed by the absorption spectra of *Synechocystis* in the wavelength range of 380–730 nm (Supplementary Fig. 10). In the absence of AuNPs, four characteristic absorption bands were observed, which corresponded to chlorophyll a at 440 nm and 680 nm, carotenoids at 480 nm, and phycobilisomes at 630 nm^40^. After incubation with AuNPs, the absorption around 480–580 nm increased significantly, which demonstrated the binding of AuNPs with *Synechocystis* cells. The narrowed difference between two strains might result by the shielding effect of photosynthetic pigments. In addition, the binding capacity of two strains was quantified by using ICP-MS. As shown in Fig. 4b, the binding amount of AuNPs by strain M6G9Δ*pilT* was approximately 47.79 ± 0.07 μg per 10^7^ cells, which was about 2.8 times higher than that of the control strain Δ*pilT*. This higher binding capacity might partially attributed to the increased negative charge resulting from the overexpression of M6G9 in strain M6G9Δ*pilT* (Fig. 3d), according to a previous study, in which a more negative value of zeta potential was favorable for the adsorption process in bacteria-AuNPs systems^41^. These results suggested that *Synechocystis* cells can naturally bind AuNPs, whereas the presence of M6G9 could dramatically improve the binding capacity.

To further investigate the enrichment of AuNPs on the cell surface of *Synechocystis*, TEM and energy-dispersive X-ray spectroscopy (EDX) elemental mapping were conducted. As shown in Fig. 4c, EDX mapping for Au and N elements revealed that AuNPs were enriched around the cell surface of *Synechocystis*. In addition, the enrichment contents of strain M6G9Δ*pilT* was more than that of the control strain Δ*pilT*, which was consistent with absorption spectra and ICP-MS analysis. It was found that most of AuNPs were specifically bound to the pili of the strain M6G9Δ*pilT* and assembled as hybrid pilus bundles (Fig. 4d and Supplementary Fig. 11). In contrast, no attachment of AuNPs to the extracellular pilus was observed for the strain Δ*pilT* (Supplementary Fig. 12). These results demonstrated that AuNPs were targeted bond to the M6G9-tagged pili due to the specific interaction between M6G9 and AuNPs. To assess the interparticle distance in the actual biohybrid electrode, we performed a scanning electron microscopy (SEM) analysis for the biofilm surface. This analysis revealed that the smallest interparticle distances of AuNPs on the surface of M6G9Δ*pilT* biofilm appeared to be less than 5 nm (Supplementary Fig. 13). It can be speculated that within the three-dimensional biofilm, where cell surfaces overlap, the effective interparticle distances would be even smaller, thus facilitating effective electron transfer and supporting the formation of a conductive network. TEM ultrathin sections confirmed that there was no any AuNPs distribution inside the M6G9Δ*pilT* cells, which excludes the possibility of AuNPs interfere with PETCs (Supplementary Fig. 14). Spot assay and quantitative colony-forming unit (CFU) counts showed no significant impact of 12-h incubation with AuNPs on the viability and growth of strain M6G9Δ*pilT* (Supplementary Figs. 15 and 16a). Furthermore, fluorescence staining using membrane-impermeable propidium iodide (PI) revealed that the integrity of cell membranes was not affected by AuNPs enrichment (Supplementary Fig. 16b). While long-term liquid culture of *Synechocystis* showed slight growth inhibition (Supplementary Fig. 17), the acute assays confirmed excellent biocompatibility for the duration of biohybrid assembly and testing.

### AuNPs enrichment improved photocurrent generation

**Fig. 5:**
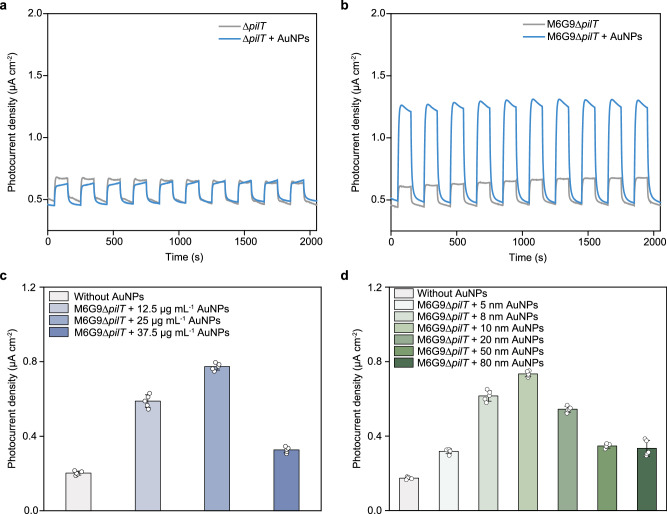
Enhanced photocurrent generation of M6G9Δ*pilT* cells by targeted enrichment of AuNPs. **a** Photocurrent density of Δ*pilT* cells in the absence or presence of AuNPs. **b** Photocurrent density of M6G9Δ*pilT* cells in the absence or presence of AuNPs. **c** Photocurrent density of M6G9Δ*pilT* cells treated with AuNPs at different concentrations. **d** Photocurrent density of M6G9Δ*pilT* cells treated with AuNPs at different sizes. Data in **c**, **d** are presented as mean values ± SD from *n* = 5 independent experiments. Source data are provided as a Source Data file.

We next measured the photocurrent generation of *Synechocystis*-AuNPs hybrids to investigate the effect of pili-localized AuNPs on EET. Photocurrent measurement was performed in a three-electrode bioelectrochemical system, in which an indium tin oxide (ITO) conductive glass was used as working electrode. Representative photo-responsive currents were observed at an applied potential of 0.7 V versus Ag/AgCl under the light/dark cycles. In the absence of AuNPs, both strain M6G9Δ*pilT* and strain Δ*pilT* produced a comparable photocurrent about 0.19 μA  cm^−2^ (Fig. 5a, b). For control strain Δ*pilT*, the treatment with AuNPs did not induce significant changes on photocurrent (Fig. 5a and Supplementary Fig. 18). However, the photocurrent density of strain M6G9Δ*pilT* after treated with AuNPs improved to 0.82 μA cm^−2^, which was about 4.3-fold higher than that of untreated cells (Fig. 5b). The greatest improvement was observed when the concentration of AuNPs solution was 25 μg mL^−1^ (Fig. 5c). Additional non-targeting controls including wild type strain and an engineered strain displaying non-binding peptide (A6G9Δ*pilT*) showed low AuNPs binding capacity and no significant photocurrent enhancement (Supplementary Fig. 19), reinforcing that the specific M6G9-AuNPs interaction is essential for the observed performance gains. It was found no significant enhancement in continuous dark current upon AuNPs treatment (Supplementary Fig. 20), which was ascribed to the depletion of intracellular reductants by basal respiration over the 4–6 h drying process, leaving a small reservoir of electrons for dark current. The small current enhancement observed in dark periods (Fig. 5b) may stem from residual photosynthetic electrons generated in the light period. Thus, the conductive pili enhanced the extraction of a high-flux photosynthetic electron stream but cannot significantly amplify a signal limited by the electron availability in the dark. Meanwhile, we observed that the biofilm formed by the strain M6G9Δ*pilT* treated with AuNPs exhibited a tendency to aggregate in the middle zone, corresponding to the fewer cells on the edge (Supplementary Fig. 21). This phenomenon might reflect the enhanced cell-cell interaction, which will be discussed in the next section. Considering the binding motif of M6G9 was very short, we speculated that the size of AuNPs might influence the interaction with pili-tagged M6G9. As shown in Fig. 5d, the 10 nm AuNPs yielded in highest photocurrent improvement, with 8 nm and 20 nm followed closely, while 5 nm, 50 nm and 80 nm provide a more modest, approximately twofold improvement. This suggests an optimal size window around 8–20 nm, possibly related to binding pocket matching. Ferricyanide was widely used as a mediator in the photocurrent generation of *Synechocystis*. The addition of ferricyanide indeed improved the photocurrent improvement of strain M6G9Δ*pilT* and strain Δ*pilT* (Supplementary Fig. 22). After treatment with AuNPs, it was found that ferricyanide could still enhance the photocurrent of strain Δ*pilT*, whereas a synergistic improvement was not observed for strain M6G9Δ*pilT*. In view of the similar photocurrent pattern for the two groups with ferricyanide, we speculated that the introduction of ferricyanide might influence the binding of AuNPs to cells. In addition, neither AuNPs alone or heat-inactivated cells treated with AuNPs did not produce any detectable photocurrent, which excludes the possibility that the increased photocurrent was contributed by the abiotic catalytic properties of AuNPs (Supplementary Fig. 23). These results demonstrated that the targeted AuNPs enrichment on cell surface significantly enhanced the photocurrent generation of *Synechocystis*, which might attribute to the enhanced interfacial electron transfer and biofilm formation.

The stability of biohybrid system was evaluated by longer-term chronoamperometry under repeated 8 h light/3 h dark cycles to simulate daily operation. The dark period was shortened to 3 h for minimizing the biofilm detachment. The biohybrid systems maintained its fourfold photocurrent enhancement over controls for 1 or 2 cycles, when light intensity was set at 400 and 200 μmol photons m^−2^ s^−1^, respectively (Supplementary Fig. 24). Subsequent photocurrent decay was observed for both hybrid and control systems, which was attributed to the cell bleaching under non-ideal growth conditions such as continuous illumination, insufficient gas exchange and mass transfer, and lack of carbon source. Additionally, photocurrent stability might be affected by the biofilm diffusion on the electrode (Supplementary Fig. 24). These results indicate the performance limit in our systems was cyanobacterial viability under current electrochemical conditions, not AuNPs detachment or aggregation.

**Fig. 6:**
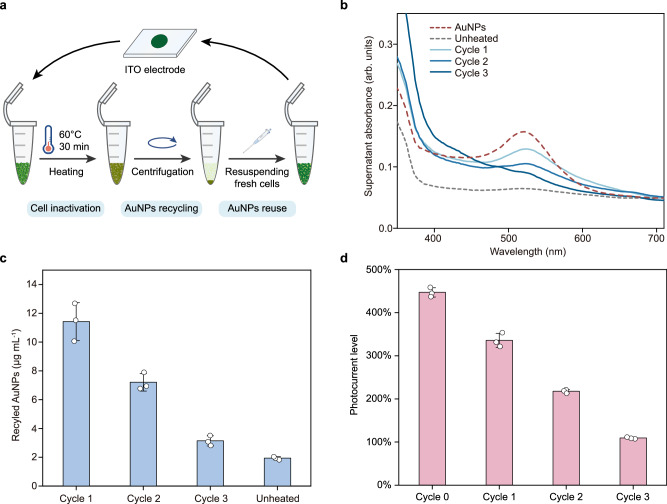
Recycling and reuse of AuNPs in photosynthetic cells. **a** Schematic illustration of the experimental process in recycling and reuse of AuNPs. After photocurrent measurement, the AuNPs decorated-cells were scraped off from the ITO electrode and the collected cells were subjected to heat treatment at 60 °C for half hour to induce cell death and AuNPs release. After centrifugation, the recycled AuNPs solution in supernatant was used to resuspend fresh cells and forming biohybrid for next-cycle photocurrent measurement. **b** The absorption spectra of recycled AuNPs solutions. The red line represents the initial AuNPs solution supplied to photosynthetic cells. **c** The concentration of recycled AuNPs solutions quantified by ICP-MS. **d** Photocurrent levels of photosynthetic cells with recycled AuNPs, in comparison to those without AuNPs. Data in **c**, **d** are presented as mean values ± SD from *n* = 3 independent experiments. Source data are provided as a Source Data file.

To investigate the long-term biological disposition of AuNPs, we perform an experiment to assess whether AuNPs could be recycled and reused by mimicking the natural regenerative process of photosynthetic cells. After photocurrent measurement, the AuNPs decorated photosynthetic cells were collected from the electrode and subjected to heat treatment to inactivate cells and desorb AuNPs (Fig. 6a). The released AuNPs solution was used to resuspend fresh photosynthetic cells and form a biohybrid for the next-cycle photocurrent measurement. As shown in Fig. 6b, the recycled supernatant showed a characteristic absorption peak of AuNPs, indicating the AuNPs deposited on photosynthetic cells will desorb into the medium once cell inactivated, although the recovery efficiency decreased along with the recycle times. Quantitative analysis using ICP-MS indicated the recovery efficiency of AuNPs was 50~65% (Fig. 6c). The rest of AuNPs remained in cell pellets due to the non-specific binding and centrifugation effect. Higher recovery rate would be benefited by treatment with peptidases to induce AuNPs release, followed by chromatographic separation or magnetic separation. Photocurrent measurements showed that the recycled AuNPs still enhanced the photocurrent generation, and the improvement ranges were positively correlated to the content of recycled AuNPs (Fig. 6d). These results demonstrated that the AuNPs deposited on photosynthetic cells could transfer from inactivated cells to the fresh cells without compromising their activity across multiple generations. We believe the primary limitation for long-term operation of the biohybrid system is cell viability and retention on the electrode, rather than the loss of nanoparticles. Design of advanced BPV structures that support cell growth and cell immobilization is essential for further development.

### Conductive and dense biofilm contributes to photocurrent improvement

**Fig. 7:**
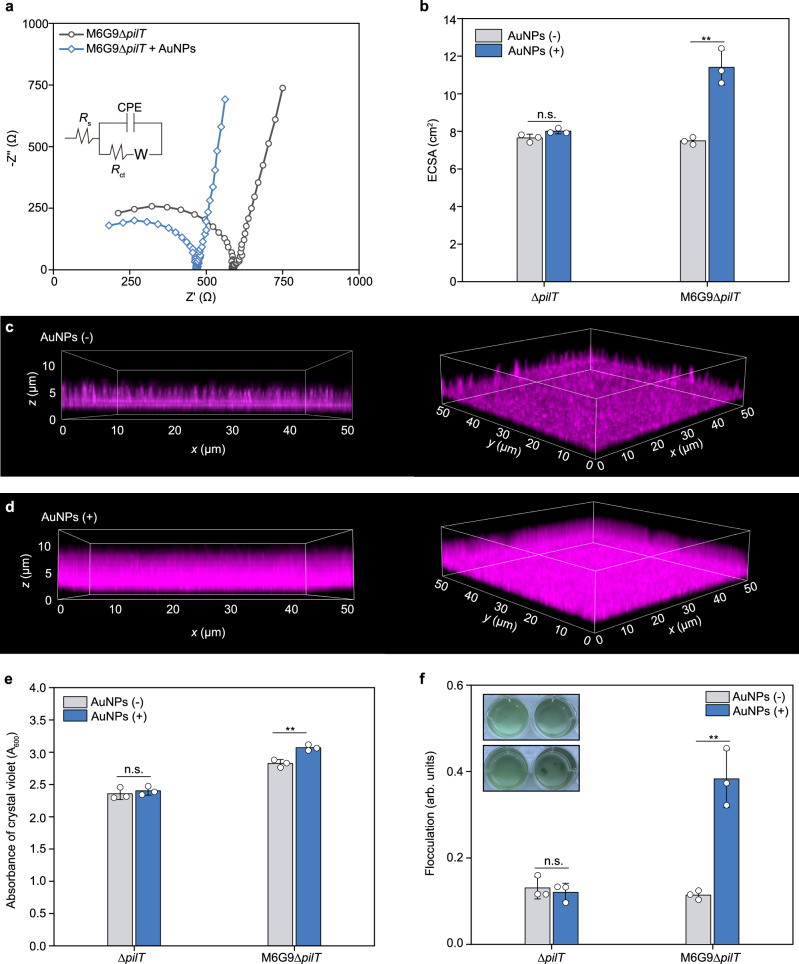
Conductive and dense biofilm contributes to photocurrent improvement. **a** Nyquist plots of the ITO electrode attached by M6G9Δ*pilT* cells in the absence or presence of AuNPs. The inset shows the equivalent electrical circuit model used to calculate the resistances. *R*_s_: solution resistance; *R*_ct_: charge transfer resistance; CPE: constant phase element; W: Warburg diffusion element. **b** The ECSA of biofilm-loaded ITO electrodes determined by CV analysis. Data are presented as mean values ± SD from *n* = 3 independent experiments. **c** CLSM images showing biofilm thickness of M6G9Δ*pilT* cells in the absence of AuNPs. **d** CLSM images showing biofilm thickness of M6G9Δ*pilT* cells in the presence of AuNPs. **e** Crystal violet assay of tightly attached cells loaded on ITO electrode. Data are presented as mean values ± SD from *n* = 3 independent experiments. **f** Flocculation assay of strains Δ*pilT* and M6G9Δ*pilT* in the absence or presence of AuNPs. Inset: photographs of flocculation assay. The top row represents strain Δ*pilT* and the bottom row represents strain M6G9Δ*pilT*. The left column represents without AuNPs and the right column represents in the presence AuNPs. Two-sided *t*-test was used to determine the significance (^**^p < 0.01, n.s. indicates no significant difference). The *p* values in **b**, **e**, **f** (from left to right) are 0.0624, 0.0020, 0.5195, 0.0047, 0.6230 and 0.0023, respectively. Data are presented as mean ± SD from *n* = 3 independent experiments. Source data are provided as a Source Data file.

Based on the aforementioned findings, we proposed that photocurrent enhancement might attribute to the improved electron transfer in cellular and biofilm levels. On the one hand, the electrically conductive AuNPs would form a conductive layer on the cell surface, thus improving the interfacial electron transfer. On the other hand, the observed cell aggregation might result in a dense biofilm, thus improving the intercellular electron transfer. To explore the role of pili-localized AuNPs in the interfacial electron transfer process, electrochemical impedance spectrometry (EIS) analysis was performed. Nyquist plots showed the well-defined semicircles over the high frequency range (Fig. 7a and Supplementary Fig. 25), and the diameter of semicircle corresponds to the charge-transfer resistance (*R*_ct_). For the strain Δ*pilT*, the *R*_ct_ values in the absence and presence of AuNPs were 539 Ω and 530  Ω, respectively. This suggested that the natural random binding of AuNPs by *Synechocystis* did not contribute to the interfacial electron transfer process. By contrast, the *R*_ct_ value of strain M6G9Δ*pilT* treated by AuNPs was 398 Ω, which was significantly lower than that of untreated cells (*R*_ct_ = 516 Ω). In addition, quantifying the electrochemically active surface areas (ECSA) of the biofilm-loaded ITO electrodes showed that AuNPs treatment significantly increased the ECSA of M6G9Δ*pilT* biofilm by 52%, while there was no effect for strain Δ*pilT* (Fig. 7b and Supplementary Fig. 26). These results implied that the pili-localized AuNPs enhanced the charge transfer rates at the cell-electrode interface.

As mentioned above, AuNPs enrichment on the pili structure induced a cell aggregation for the strain M6G9Δ*pilT*. It was hypothesized that the variation in the morphology of the biohybrid on the electrode might reflect an alteration in the cell-electrode and cell-cell interactions^42^. We first performed a detailed characterization of biofilm structure using CLSM based on chlorophyll fluorescence. The analysis revealed that AuNPs enrichment induced a thicker and denser biofilm in the central zone of the electrode. Without AuNPs, cells are pushed to the edge of the droplet due to surface tension during the drying process (Supplementary Fig. 21). The AuNPs-enhanced cell-cell interaction prevented this migration to the edge, leading to a uniform cell layer with improved effective interfacial area. This structural change directly explains the observed increase in ECSA and the correlated decrease in charge-transfer resistance, as a larger population of cells was effectively wired to the electrode. To investigate the cell-electrode interaction of the biofilm on ITO electrode, the biofilm was washed with BG11 medium three times, the residual cells that tightly attached onto the electrode were dyed with crystal violet. It was shown that the treatment of AuNPs induced more M6G9*ΔpilT* cells tightly attached onto the electrode, while it did not augment the biofilm formation for the strain Δ*pilT* (Fig. 7e). The microscopic images also showed that the amount of tightly attached cells of strain M6G9Δ*pilT* was more than that of strain Δ*pilT* (Supplementary Fig. 28). These findings suggest that pili-localized AuNPs induced stronger cell-electrode interaction, thus might contribute to photocurrent improvement. To be sure, the difference in biofilm formation for strains M6G9Δ*pilT* and Δ*pilT* in the absence of AuNPs did not induce any photocurrent changes (Figs. 5a, b and 7e). This could be explained as that the low interfacial electron transfer in the absence of AuNPs did not be able to give a response for the variation in biofilm formation.

The cell-cell interaction was further evaluated using a flocculation assay. The aggregation was occurred when strain M6G9Δ*pilT* was incubated with AuNPs for 2 h (Supplementary Fig. 29). The flocculation was quantified by measuring the pixel intensity in photographs, where a higher pixel intensity indicates increased flocculation^43^. As shown in Fig. 7f, the strain M6G9Δ*pilT* treated with AuNPs exhibited a significant increase in flocculation when compared with untreated cells. In contrast, no aggregation was observed for the strain Δ*pilT*, regardless of the absence or presence of AuNPs. SEM analysis showed that the compact cluster was formed for strain M6G9Δ*pilT* after treatment with AuNPs, while the cells of strain Δ*pilT* were distributed scatteredly (Supplementary Fig. 30). However, AuNPs treatment did not alter the zeta potential of *Synechocystis*, regardless of wild-type or different engineered strains (Supplementary Fig. 31), suggesting that the enhanced cell-cell interaction was not induced by the electrostatic effect. These results demonstrated that the pili-localized AuNPs did not only enhanced the interfacial interaction between cells and the electrode, but also cell-cell interaction. Cell-to-cell binding might be beneficial to the electron transfer of distal cells that not directly contacted with the electrode when AuNPs conductive layer was formed on a single cell. That is, the conductive layer on one cell will contacted with the conductive layers of surrounding cells, thus forming a compact conductive network in intercellular spaces. This conductive network collected electrons exported from all loading photosynthetic cells and transported them to the electrode side.

Based on the above findings, photocurrent enhancement is not due to a single factor but a synergistic combination of improved interfacial charge transfer and increased effective cell loading. At the interface, conductive pili increased the ECSA by 52% and decreased the charge-transfer resistance by 23%. In the biofilm, AuNPs enrichment doubled the thickness of the electroactive biofilm through enhanced intercellular interaction. Moreover, the AuNPs conductive network facilitated the effective electron transfer inside the dense biofilm. In short, AuNPs enrichment doubled both the number of effectively wiring cells and electron transfer efficiency, ultimately improving the photocurrent density by approximately fourfold.

## Discussion

This study establishes a synthetic biology framework for programming conductive interfaces on living photosynthetic cells. By genetically fusing a high-affinity GBP (M6G9) to type IV pili, we engineered a scalable biological scaffold that directs the assembly of gold nanoparticles (AuNPs) into a cohesive, electron-conducting network. The gold binding peptide M6G9 was densely displayed on the surface of the pili network once the *pilT* gene was knockout. The M6G9-fused hyperpiliated strain exhibited a significantly improved capacity in enrichment of AuNPs compared to the control strain. This targeted approach resulted in a fourfold enhancement in photocurrent generation of *Synechocystis*. It was demonstrated that the enrichment of AuNPs formed a conductive layer in the cells-electrode interface, thus reducing the interfacial electron transfer resistance. Furthermore, the enrichment of AuNPs on the cell surface promoted biofilm formation, thus further facilitating the electron collection from photosynthetic cells. These findings provide a strategy for the rational design of conductive surfaces on the cell envelope of photosynthetic microorganisms, offering a path towards to overcome the limitations associated with inefficient extracellular electron extraction from PETCs.

Our work represents a significant advancement over previous biohybrid strategies, which have largely relied on the stochastic adsorption of nanomaterials. Such non-specific interactions lead to random, inefficient distribution and high material consumption^44–48^. Moreover, these nonspecific interactions are practically material-inefficient, i.e., it often needs much more materials. Kuruvinashetti et al.^46^ translocated pre-fabricated AuNPs into the cell wall-less *Chlamydomonas reinhardtii*, showing an improvement in maximum power by 15%. Liu et al.^48^ achieved in vivo synthesis of AuNPs inside *Synechocystis* through the biological reduction of Au^3+^ solution, resulting in a 33-fold improvement in maximum power density. The calculated requirement for Au material in above two systems were 6.4 × 10^−15 ^mol/cell and 1.9 × 10^−14 ^mol/cell, respectively (Supplementary Table 5). In this study, the requirement for Au material was 9.4 × 10^−17 ^mol/cell, which represents two orders of magnitude less AuNPs loading than non-targeted methods. Most importantly, we demonstrate that non-targeted AuNPs binding, as seen in our control strain (*∆pilT*), fails to enhance photocurrent, underscoring that spatial precision is critical for forming an effective electron conduit. The stochastically distributed nanomaterials might not be able to form a conductive network, thus hindering their conductive properties. By contrast, the targeted enrichment of AuNPs on a specific cellular structure enabled the formation of well-defined metal conduits and conductive networks, thereby facilitating the EET of individual cells. This approach marks a significant conceptual advance, as it transitions from passive decoration to active, programmable assembly, enabling the creation of well-defined nano-bio interfaces that optimize electron flow. The targeted enrichment of AuNPs on pili structure represents a proof-of-concept that rational construction of conductive layers on the cell envelope.

When contextualized within the broader field of BPV, our mediator-free system achieves a photocurrent density of 0.82 μA cm^−2^, which is among the highest reported for a planar ITO electrode (Supplementary Data 1). Abiotic engineering approaches were mostly used to enhance electron transfer at biotic–abiotic interface in previously reported BPV systems^6^. This includes electrode engineering, device miniaturization and electron mediator deployment. Among them, the most significant performance improvements came from electrode design. ITO-coated glass was most popularly used in BPV studies due to its low cost, good electrical conductivity and optical transparency, but the high photocurrent outputs came from the non-planar architectures such as microporous ITO, micropillar ITO and 3D conductive gels. To date, the highest photocurrent density was 245 μA cm^−2^, which was achieved by using 2,6-dichloro-1,4-benzoquinone (DCBQ) mediator and a branched micropillar made by ITO nanoparticles (BP-ITO)^49^. This BP-ITO electrode exhibited excellent cell loading, light utilization and electroactive surface than flat ITO. Besides electrode engineering, exogenous electron mediators, such as DCBQ and ferricyanide, could improve the instant photocurrent levels by several to hundreds of fold. For example, the photocurrent even for BP-ITO was decreased to 1.93 μA cm^−2^ when mediator DCBQ was excluded. However, the cytotoxicity and photochemical instability of electron mediators severely restrict the long-term performance of BPV systems. It should be noted that the highest photocurrent of 245 μA cm^−2^ only represents the initial photocurrent in the first light cycle due to it dropped rapidly under mediated condition. Our strategy fundamentally enhances the electron-transfer capability of the biological component itself. This “engineered biocatalyst” approach is orthogonal to, and can be synergistically combined with, advances in electrode engineering, paving the way for next-generation, high-performance BPV devices.

The choice of type IV pili as a scaffold was instrumental to our success. As self-assembling, polymeric structures, pili provide a dense, organized surface for functional peptide display that operates across cellular and population levels. The intrinsic self-organizing properties of pili function not only at the single-cell level but also at the population level, enabling the creation of scaffolds with biomineralizing and cross-linking functionalities, thereby facilitating the development of controllable and tunable systems. In this study, the fusion of the M6G9 peptide with PilA1 significantly enhanced cell-cell interactions through the subsequent assembly of AuNPs. In contrast, other outer membrane proteins, such as eCPX or LPP-OmpA did not show similar effects^50–52^. The difference in spatial arrangement and self-organizing ability of these scaffolds underscores the advantages of using self-assembling biomolecules as scaffolds to construct hybrid systems. In addition, the expression of M6G9 in the hyperpiliated strain significantly increased the magnitude of zeta potential, which enables cells stay in suspension in favor of harvesting light energy. These advantages allow type IV pili to serve as a structural building block for more applications, such as biosensing^53^ and water treatment^54^, and highlight the importance of leveraging dynamic endogenous biopolymers over static surface proteins for constructing complex hybrid systems with emergent properties.

Constructing microorganisms-material hybrid systems were regarded as an artificial organism evolution strategy, which enables microorganisms to be functionalized with synthetic materials^55^. Metal transmembrane pathway serves as an alternative for a complete biological electron conduit, transforming cyanobacteria into electroactive bacteria and thereby maximizing photoelectric conversion efficiency. The future bottom-up design of controllable transmembrane metal conduits requires broadening the range of programmable building blocks and nanomaterials available in photosynthetic microorganisms. This can be achieved by rationally combining tools from materials science, structural biology and synthetic biology. Machine-learning-guided protein design could yield a suite of binding peptides for diverse nanomaterials, while the identification of other cellular anchor proteins would allow for the construction of multi-layered conductive pathways spanning the entire cell envelope. We envision such programmable biohybrids advancing beyond energy harvesting to applications in environmental sensing, bioremediation, and sustainable biochemical production.

## Methods

### Culture conditions

All strains used in this study were listed in Supplementary Table 2. The cyanobacterial strains were cultured in BG11 medium supplemented with 50 mM NaHCO_3_ at 30 °C. The light intensity applied in cyanobacterial cultures was 150  μmol photons m^−2^ s^−1^. Antibiotics at a final concentration of 30 μg mL^−1^ for chloromycetin, 50 μg mL^−1^ for spectinomycin, and 20 μg mL^−1^ for gentamycin were added to the BG11 medium when required. *E. coli* strains were cultivated in Luria Broth (LB) medium at 37 °C. Cell optical densities at 730 nm (OD_730_) for cyanobacteria and at 600 nm (OD_600_) for *E. coli* were measured using a TU-1900 UV-VIS spectrophotometer (Persee, Beijing, China). To exploring the cytotoxicity of AuNPs, AuNPs were supplied into the liquid cultures at different concentrations during the inoculation stage.

### Strains construction

The plasmids and primers used in this study were listed in Supplementary Tables 3 and 4. To generate the deletion/replacement mutants in *Synechocystis*, a suicide plasmid carrying the insert and homologous fragments flanking editing site was required. Taking the *pilA1* deletion as an example, the primers *pilA1* up-F/*pilA1* up-R and *pilA1* down-F/*pilA1* down-R were used to amplify the upstream fragment and downstream fragment of *pilA1*. The primers Cm-F/Cm-R were used to amplify the insert fragment encoding spectinomycin resistance. These three fragments were fused and inserted into the pUC57 backbone using a Gibson assembly kit (E5510S, New England BioLabs). The final construct pUC-Δ*pilA1* was introduced into the *Synechocystis* cells through natural transformation or electrotransformation. The transformants were obtained by selection on BG11 agar plates. The genotypes of the transformants were verified by colony PCR. For fusion expression of M6G9 with PilA1 in *Synechocystis*, the insert fragment also included the promoter P_cpc560_, *pilA1* and the coding sequence of M6G9, beyond that homologous fragments and resistance genes. To construct *E. coli* strains, the expression plasmids pET30a-GFP and pET30a-GBP were constructed using Gibson assembly method, and then introduced into *E. coli* BL21(DE3) for surface display of GFP and GBPs.

### Analytical methods

The absorbance spectra of AuNPs and *Synechocystis*-AuNPs hybrids were measured using a microplate reader Infinite M200 (TECAN, Switzerland). Zeta potentials of *Synechocystis* cells dispersed in BG11 were measured using Zetasizer Nano ZS (Malvern Panalytical Ltd, UK). The binding amounts of AuNPs were quantified by ICP-MS (Perkin Elmer, USA). The cellular viability of *Synechocystis* in the presence of AuNPs was determined by spot assay. Briefly, the cells were adjusted to an OD_730_ of 1.0 (dilution factor 0). Subsequently, tenfold serial dilutions were prepared in BG11 medium, followed by dropping 10 μL of each dilution onto BG11 agar plates. The plates were incubated at 30 °C under light for colony formation and CFUs were enumerated based on the spots. Cell membrane integrity was evaluated by fluorescence staining using 30 μM PI. Fluorescence intensity was measured at an excitation wavelength of 485 nm and an emission wavelength of 630 nm. The dead cells treated by 70% isopropanol were used as the positive control. The chemical interaction between AuNPs and M6G9 was characterized by XPS (ESCALAB 250Xi, Thermo Fisher Scientific, USA).

### Functional validation of surface display systems

For protease accessibility assay, the *E. coli* cells with surface-displayed eGFP or cytosolic-expressed eGFP were suspended in PBS buffer at pH 7.0 and the OD_600_ was adjusted to 5.0. The cells were treated with 1 mg mL^−1^ pronase (53702, Calbiochem) under 37 °C for 1 h. The GFP fluorescence intensities for treated cells and untreated cells were measured. For fluorescence-activated cell sorting analysis, the *E. coli* cells were washed three times using flow cytometry staining buffer (AFC060, Beyotime) and then incubated with AF647-conjugated anti-GFP antibody (AC1432, Beyotime, 1:20 dilution) for 1.5 h at room temperature, followed by washing to remove unbound antibody. Flow cytometry analysis was performed on a BD influx cell sorter (BD Biosciences, USA).

### Determining the binding capacity of GBPs

*E. coli* strains with surface display of GBPs were cultured in LB medium overnight, followed by dilution of 100-fold in fresh LB medium. When an OD_600_ of 0.4–0.6 was reached, the culture was induced by the addition of 0.1 mM IPTG and grown for another 12 h. The induced cells were harvested by centrifugation at 4000 × *g* for 10 min. The precipitated cells were washed thrice with 10 mM phosphate-buffered saline (PBS, pH =  7.4). The cells were resuspended in PBS, and the OD_600_ was adjusted to 1.0. The cells were then incubated with AuNPs at a concentration of 15 μg mL^−1^ under a gentle agitation for 3 h. The pellet containing cells and AuNPs was collected by centrifugation at 2000 × *g* for 10 min and washed thrice with deionized water. The binding capacity of cells for AuNPs was evaluated by ICP-MS analysis.

### Determining the binding affinity using ITC

To assess the binding affinity of the peptides M6G9 and A6G9 to AuNPs, ITC measurements were performed in an isothermal titration calorimeter (Affinity ITC LV, TA Instruments, USA). The peptides were synthesized by GenScript Biotech Corporation. AuNPs and M6G9 solutions were prepared in 10% dimethyl sulfoxide (DMSO). A 200 μL of AuNPs solution was loaded into the sample vessel. Subsequently, 50 μL of M6G9 solution was titrated into the vessel via a syringe in twenty increments with the stirring speed maintained at 600 × *g*. The temperature was kept at 25 °C. The dilution heat of M6G9 in the 10% DMSO solvent, which was obtained by titrating M6G9 into pure 10% DMSO, was accounted for and subtracted during data processing. The binding parameters were determined by fitting the ITC curves using NanoAnalyze software (TA Instruments, USA).

### Immunoblotting analysis

The cyanobacterial cultures in exponential phase were harvested by centrifugation and washed with 10 mM PBS, pH 7.4. The harvested cells were resuspended in 0.4 mL of lysis buffer (pH 8.0) containing 50 mM Tris-HCl, 150 mM NaCl, 1% Triton X-100, 1 mg mL^−1^ lysozyme, and EDTA-free protease inhibitor and incubated at 37 °C for half an hour. Subsequently, adding 0.4 mL of acid-washed glass beads and vigorously shaking the mixture for 2 min using Tissuelyser. The cell debris was removed by centrifugation. The protein concentration in the supernatant was determined using Bicinchoninic Acid (BCA) protein assay kits (P0010S, Beyotime). Proteins were separated by SDS-PAGE, followed by transferring onto a 0.22 μm polyvinylidene difluoride (PVDF) membrane. The target protein was labeled with a primary rabbit polyclonal antibody against PilA1 (Abclonal, 1:3000 dilution), followed by labeling using a secondary Goat-anti Rabbit-HRP antibody (BE0103, Easybio, 1:3000 dilution).

### Assembly of AuNPs on *Synechocystis*

The citrate-stabilized AuNPs with average size of 10 nm were obtained from 3ABio (Anhui, China). The cyanobacterial cultures grown for 3 d were harvested by centrifugation at 5000 × *g* for 5 min and washed twice with fresh BG11 medium that free of ammonium ferric citrate. The *Synechocystis* cells were concentrated to an OD_730_ of 25. A certain amount of AuNPs was added into 200 μL of concentrated *Synechocystis* cells. The mixture of *Synechocystis* cells and AuNPs was incubated for 2 h under light with an intensity of approximately 30 μmol photons m^−2^ s^−1^. The formed *Synechocystis*@Au hybrids were harvested by centrifugation at 5000 × *g* for 5 min. The absorbance spectra of residual AuNPs in supernatant were measured. The *Synechocystis*@Au hybrids were resuspended in fresh medium for TEM/SEM analysis and photocurrent measurement.

### Electrochemical characterizations

A three-electrode bioelectrochemical system was used for photocurrent measurement and EIS analysis, including an ITO conductive glass as working electrode, a platinum wire as counter electrode and an Ag/AgCl as the reference electrode. The BG11 medium that free of ammonium ferric citrate was used as the electrolyte. A monochromatic red light (*λ* = 658 nm) with an intensity of 400 μmol photons m^−2 ^s^−1^ was applied to cyanobacterial cells. A 200 μL pre-incubated mixture of *Synechocystis* cells and AuNPs was dropped onto an ITO conductive glass and allowed to air-dry at room temperature without any immobilization. Upon dried, 20 mL electrolyte was slowly perfused into the reactor using a peristaltic pump at 0.4 mL min^−1^ to minimize detachment of cells from the electrode. The air-dried biofilm occupied a geometrical surface area of 2.1 cm^2^. Photocurrent measurements were performed under chopped light conditions (100 s light/100 s dark cycles). The applied potential of the working electrode was controlled at 0.7 V versus Ag/AgCl using a potentiostat (CHI1030C, CH Instruments, China). For photocurrent measurement of metabolically inactive cells, the cells were treated at 60 °C for 30 min and dropped onto ITO electrode as the same procedure. Abiotic photocurrent was measured using AuNPs alone dropped onto the ITO electrode. EIS measurement was conducted using a CHI660E electrochemical workstation (CH Instruments, Shanghai, China) at open circuit potential over an AC frequency range of 100 kHz to 1 mHz, with a sinusoidal perturbation of 5 mV. Cyclic voltammetry (CV) measurements at scan rates of 1, 2, 5, 10, 15, 20, and 25 mV s^−1^ were conducted to determine the ECSA. The applied potential was ranged from 0.1 to 1.0 V versus Ag/AgCl. The peak currents (*I*_p_) at each scan rate were extracted and plotted as a function of the square root of the scan rate (*ν*^1/2^). A bare ITO electrode with known surface area was used to calculate the exact ECSA values following the Randles-Sevcik equation.

### Recycling and reuse of AuNPs

After photocurrent measurement, the M6G9Δ*pilT* cells decorated with AuNPs were scraped off from the ITO electrode and suspended in fresh BG11 medium. The cell suspension was subjected to heat treatment at 60 °C for 30 min to induce cell death and AuNPs desorption. The released AuNPs were separated from dead cells by centrifugation at 2000 × *g* for 3 min. Subsequently, the recycled AuNPs in supernatant was used to resuspend fresh cells and form biohybrid for next-cycle photocurrent measurement. The concentrations of recycled AuNPs were quantified by ICP-MS.

### Flocculation assay

The cell-cell interaction was characterized using a flocculation assay as previously reported^16^. The cyanobacterial cultures in exponential phase were harvested by centrifugation and resuspended in BG11 medium at an OD_730_ of 0.5. A 2.5 mL of cell suspension was transferred into the wells of 12-well plate (3513, Corning, United States). Then, the plate was gently shaken under illumination of 30 μmol photons m^−2^ s^−1^. After incubation for 48 h, the plates were visually inspected and photographed for documentation. Images were processed for a quantitative measurement of flocculation.

### TEM and SEM analysis

The enrichment of AuNPs on the pili was observed by TEM. The samples were prepared by drop-casting the cells onto copper mesh. TEM images were recorded using an electron microscope (JEM-1200EX, JEOL, Japan) at an accelerating voltage of 200 kV. The elemental analysis was conducted with an EDX system (EDAX, AMETEK) configured to the electron microscope. To observe the phenotypes of the mutants of Δ*pilA* and Δ*pilT*, the cells were negatively stained with 0.5% uranyl acetate before drop-cast. Cross-sectional slices of *Synechocystis* cells treated with AuNPs were subjected to TEM observation to investigate the intracellular distribution of the nanoparticles.

The aggregation of *Synechocystis*@Au hybrid was observed by SEM using an SU8010 field emission electron microscope (Hitachi, Japan) at an accelerating voltage of 3 kV. The *Synechocystis* cells were initially fixed overnight with 2.5% glutaraldehyde solution. Subsequently, the cells were chemically dehydrated using the ethanol solutions at gradient concentrations of 50%, 70%, 85%, 95% and 100%. After dehydration, the samples were dried in the vacuum chamber and finally sputter-coated with gold for SEM imaging. To observe the distribution of AuNPs on cell surface, the biofilms formed on ITO electrode were directly subjected for SEM analysis using a GeminiSEM 300 scanning electron microscope (ZEISS, Germany) at an accelerating voltage of 20 kV.

### Biofilm formation assay

To characterize the biofilm structure, the biofilms on ITO electrode were analyzed by CLSM using N-SIM S Super Resolution Microscope (Nikon Instruments Inc., Japan) based on chlorophyll fluorescence. The excitation wavelength was set at 620 nm and the emission wavelength ranges from 650 to 750 nm. The z-stack imaging was performed to resolve the three-dimensional structure of the biofilm. A modified crystal violet assay was used to characterize the interaction between biofilm and ITO electrode^56^. The *Synechocystis* cells were dropped onto 1 × 1 cm ITO electrode and allowed to air-dry. Subsequently, the cell-loaded ITO electrode was subjected into the wells of 24-well plate and gently washed thrice with BG11 to remove the non-adherent cells. A 500 μL of 0.1% w/v crystal violet solution was pipetted into the well and allowed for incubation for 15 min at room temperature. The residual crystal violet was washed off with deionized water. Afterward, a 1 mL of DMSO was added into the well to release the bound crystal violet. The absorbance of the crystal violet solution in DMSO was measured at 600 nm, which was an indicator reflecting the amount of cells that bound to the ITO glass. The bound cells on electrode were imaged by CLSM.

### Ethics

This study does not involve experiment involving animals, human participants, or clinical samples

### Reporting summary

Further information on research design is available in the Nature Portfolio Reporting Summary linked to this article.

## Supplementary information

Supplementary Information

Description of Additional Supplementary Files

Supplementary Data 1

Reporting Summary

Transparent Peer Review file

## Source data

Source data

## Data Availability

All data supporting the findings of this study are available within the article and its supplementary files. Any additional requests for information can be directed to and will be fulfilled by the corresponding authors. Source data are provided with this paper.
