# Supplementary material for: Engineered conductive pili enable high-efficiency photosynthetic electron extraction in biophotovoltaics
Source: Nat Commun. 2026 Apr 25;17:5724. doi: 10.1038/s41467-026-72407-7 (PMC13323719; doi:10.1038/s41467-026-72407-7)
Supplement: Supplementary file 1 — Supplementary Information [file 41467_2026_72407_MOESM1_ESM.pdf]

Supplementary Information

Engineered conductive pili enable high-efficiency photosynthetic electrons extraction in biophotovoltaics

Wang *et al*.

Supplementary Figures

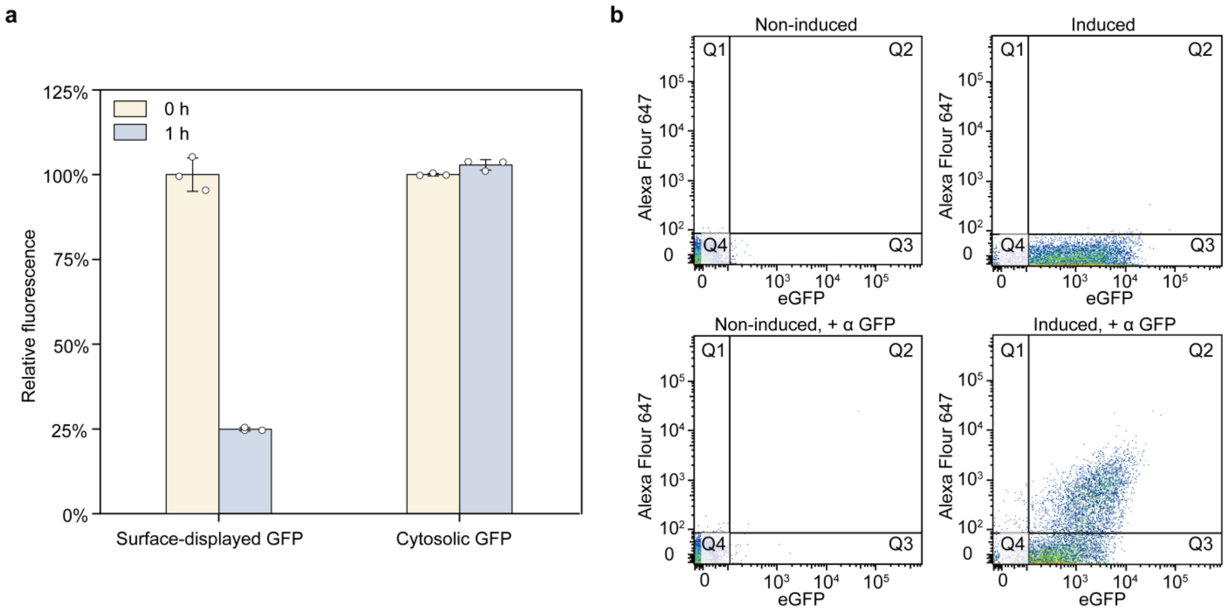

Supplementary Fig. 1. Characterization of surface-displayed eGFP. a, Fluorescence intensity of *E. coli* cells before and after proteinase shaving for 1 h. Data are presented as mean values ± SD from n = 3 independent experiments. **b**, Flow cytometric analysis showed that only *E. coli* cells with surface-displayed eGFP could be labeled by anti-eGFP antibody. Source data are provided as a Source Data file.

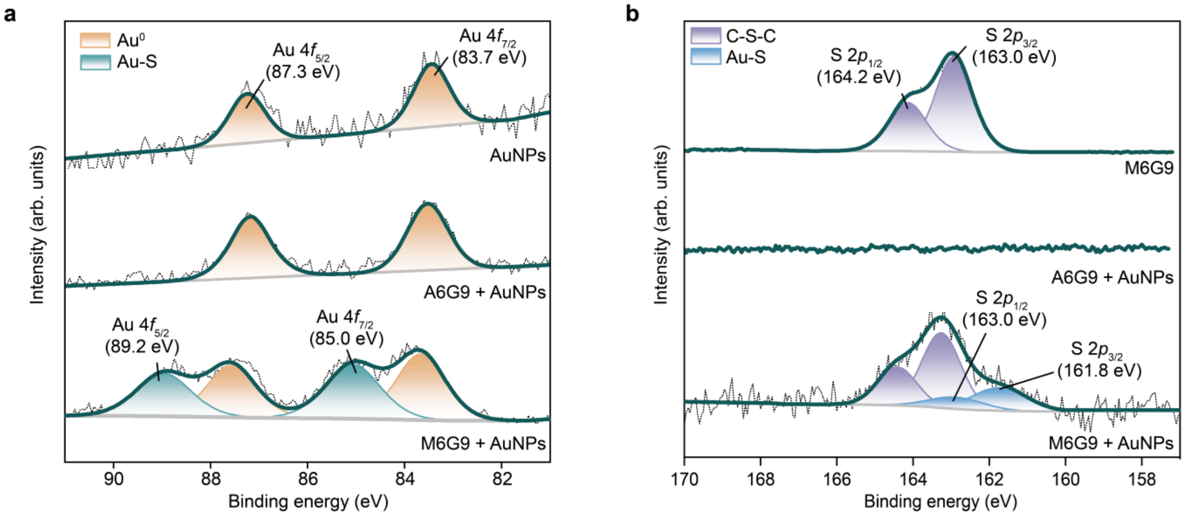

Supplementary Fig. 2. XPS characterization of chemical interaction between AuNPs and M6G9. a, XPS spectra of Au 4*f*. **b**, XPS spectra of S 2*p*. Source data are provided as a Source Data file.

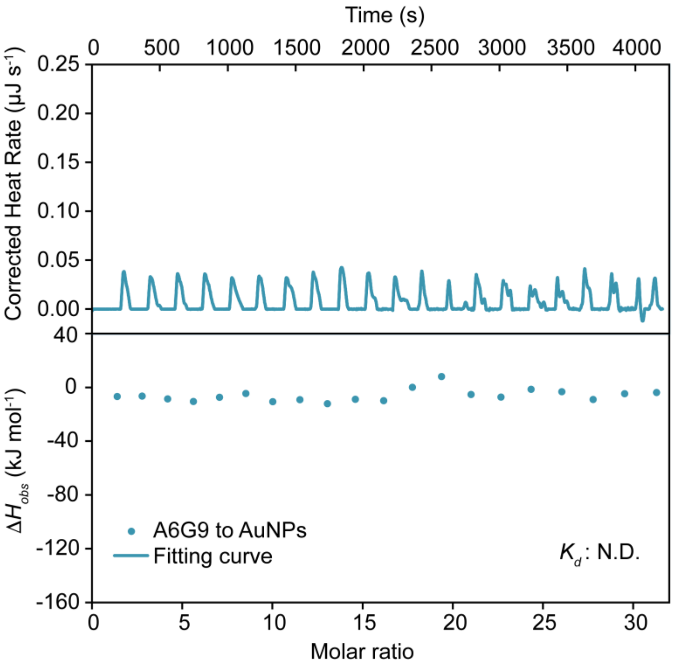

Supplementary Fig. 3. Isothermal titration calorimetry (ITC) analysis of the interaction between AuNPs and A6G9. Source data are provided as a Source Data file.

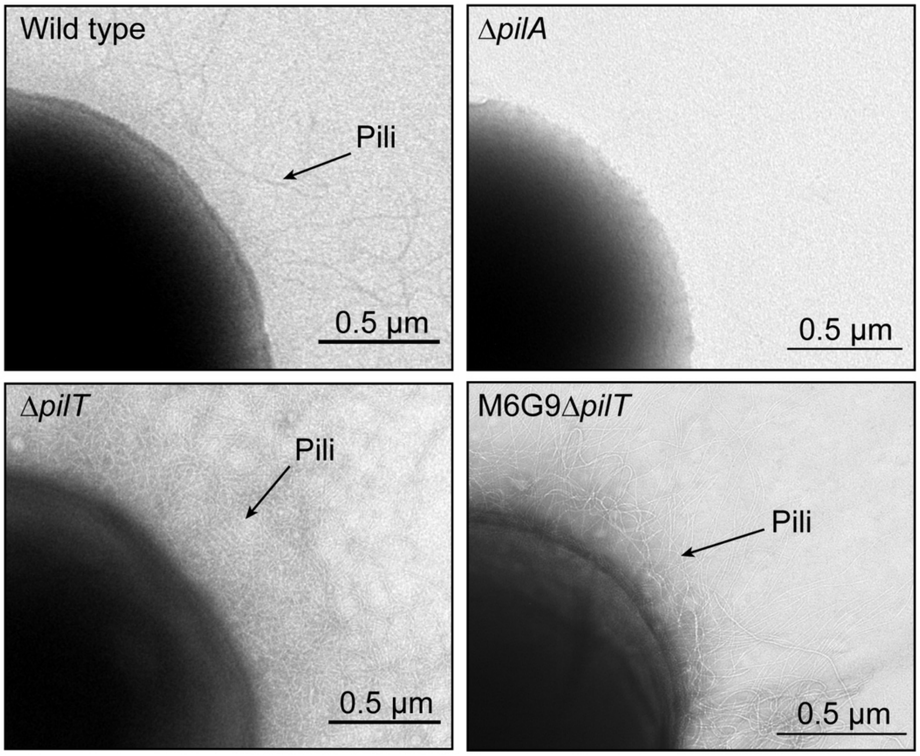

Supplementary Fig. 4. Negative-staining TEM images of wild-type and engineered *Synechocystis* strains. The experiments were repeated three times independently with similar results. Source data are provided as a Source Data file.

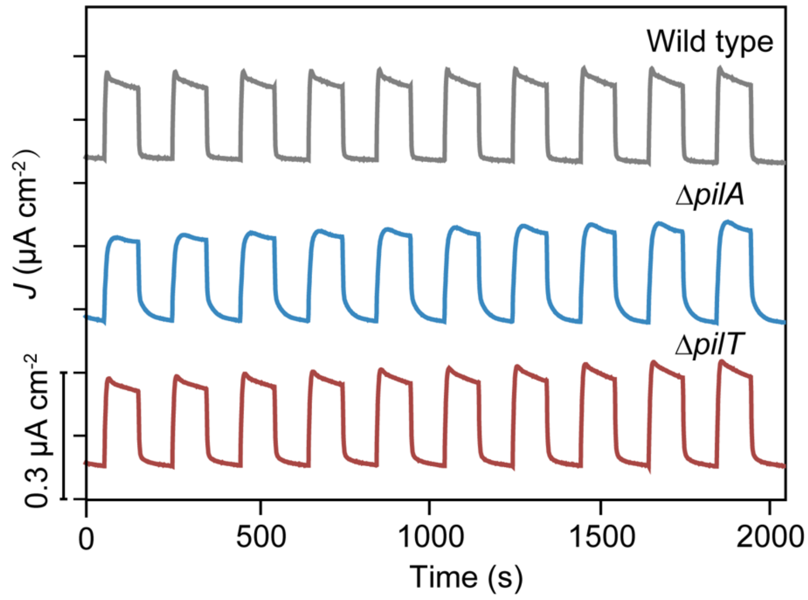

Supplementary Fig. 5. Photocurrent generated by wild type (gray line), Δ*pilA* mutant (blue line) and Δ*pilT* mutant (red line). Source data are provided as a Source Data file.

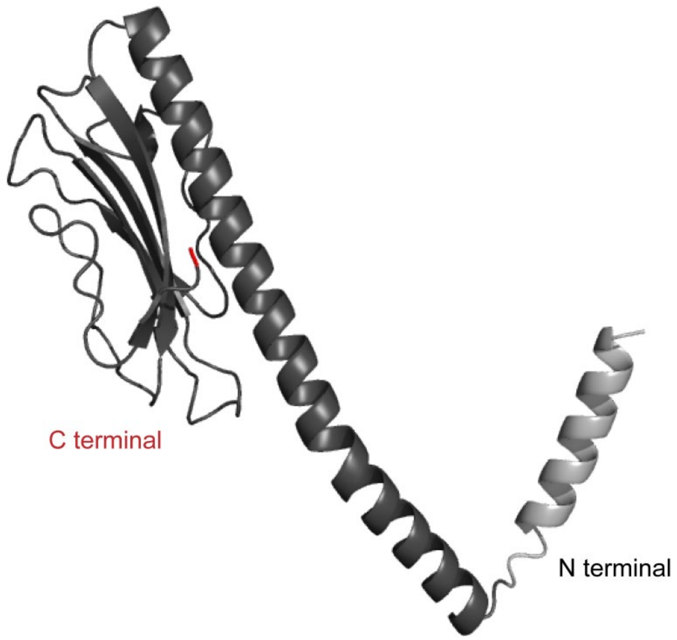

Supplementary Fig. 6. The structure of PilA1 subunit predicated using AlphaFold.

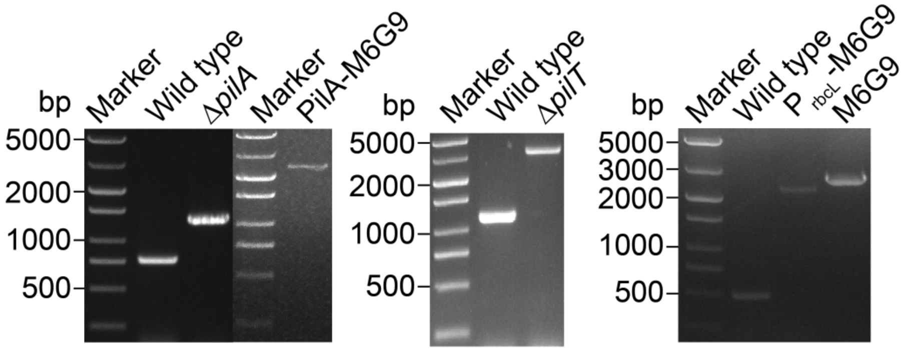

Supplementary Fig. 7. Verification of the integration of chimera genes and the knockout of targeted genes in *Synechocystis* by colony PCR with gene-specific primers. The primers used: PilA-F/PilA-R (left), PilT-F/PilT-R (middle), NS1-F/NS1-R (right). Source data are provided as a Source Data file.

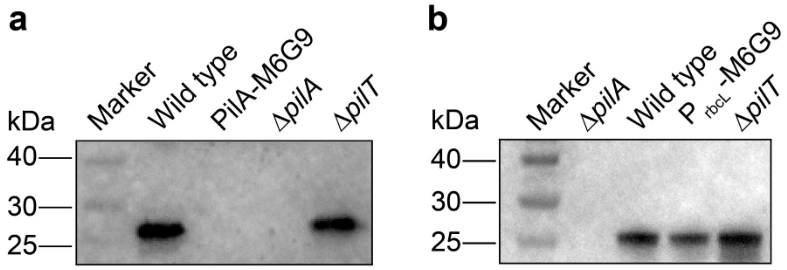

Supplementary Fig. 8. Immunoblotting analysis of whole-cell extracts of different *Synechocystis* strains. a, The *pilA1* site serves as the integration site to replace native *pilA1* with the PilA-M6G9 fusion cassette. **b**, PilA-M6G9 fusion cassette under the weak promoter P_{rbcL} was integrated into a neutral site I (NSI). Source data are provided as a Source Data file.

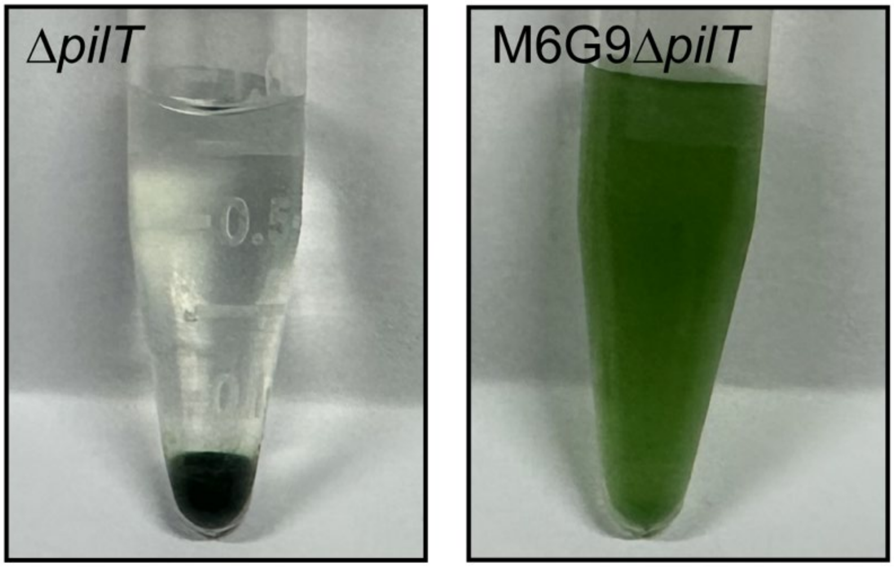

Supplementary Fig. 9. The photographs of strain Δ*pilT* and strain M6G9Δ*pilT* after standing for 12 h. A significant aggregation and sedimentation were observed for strain Δ*pilT*, whereas the strain M6G9Δ*pilT* in the solution exhibited a phenotype of suspension without sedimentation. The experiments were repeated three times independently with similar results. Source data are provided as a Source Data file.

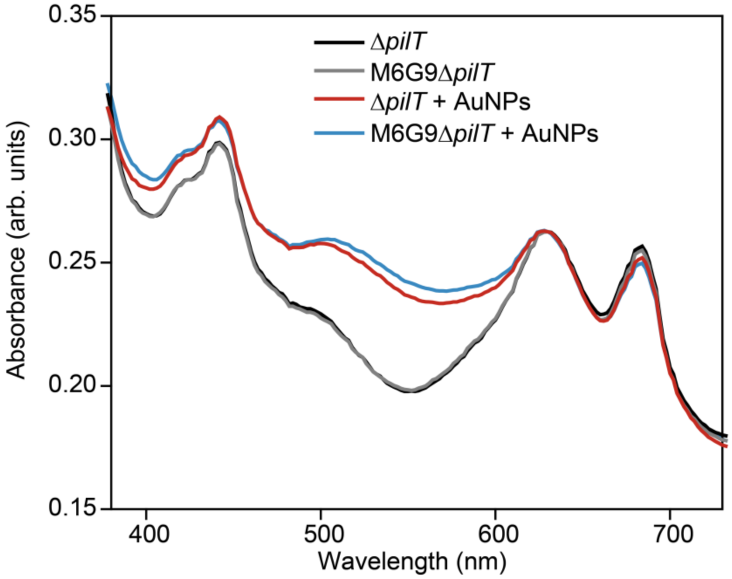

Supplementary Fig. 10. The absorption spectra of Δ*pilT* and M6G9Δ*pilT* cells treated with or without AuNPs. Four characteristic absorption bands correspond to chlorophyll a (440 nm and 680 nm), carotenoids (480 nm) and phycobilisomes (630 nm). The absorption increases around 480-580 nm indicated the binding of AuNPs by *Synechocystis* cells. Source data are provided as a Source Data file.

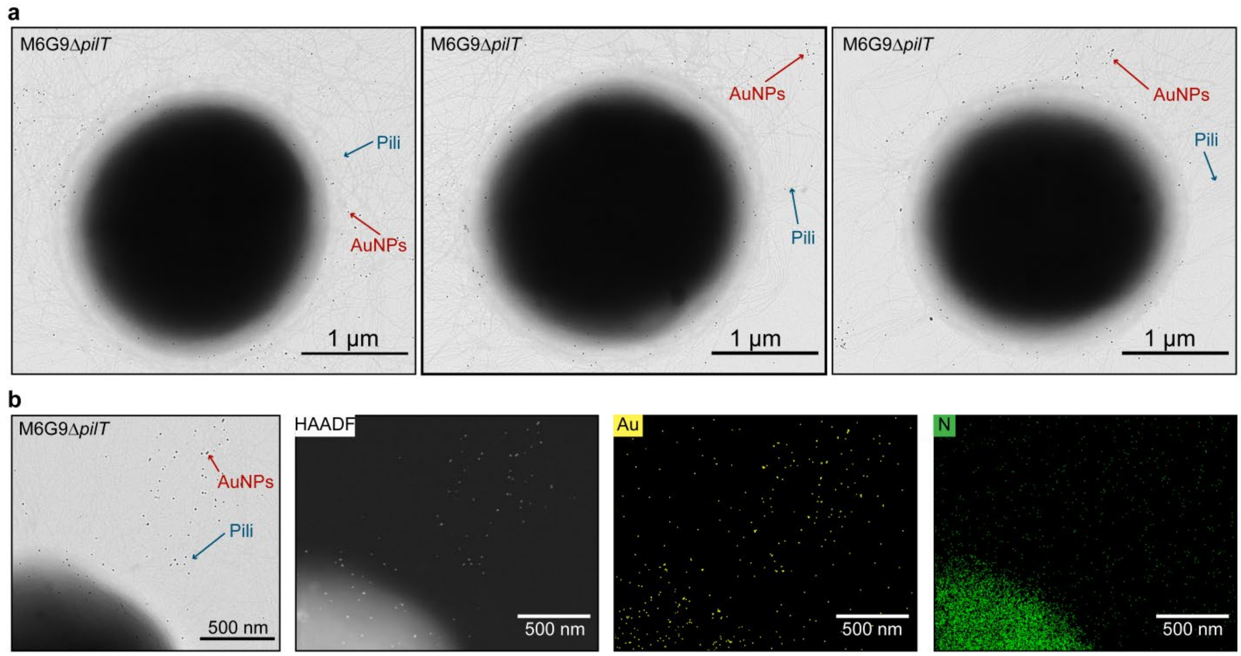

Supplementary Fig. 11. Characterization of *Synechocystis*-AuNPs hybrids. a, Three individual cells show AuNPs were bound to the pili structure of strain M6G9Δ*pilT*. **b**, The EDX mapping indicated the binding particles were AuNPs. The yellow and green represent Au atoms and N atoms, respectively. The experiments were repeated three times independently with similar results. Source data are provided as a Source Data file.

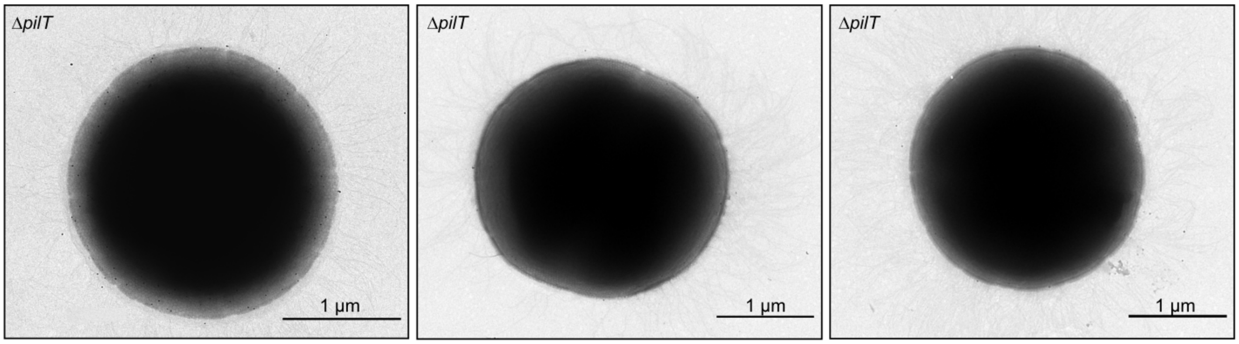

Supplementary Fig. 12. Three individual cells of strain Δ*pilT* after incubated with AuNPs. The experiments were repeated three times independently with similar results. Source data are provided as a Source Data file.

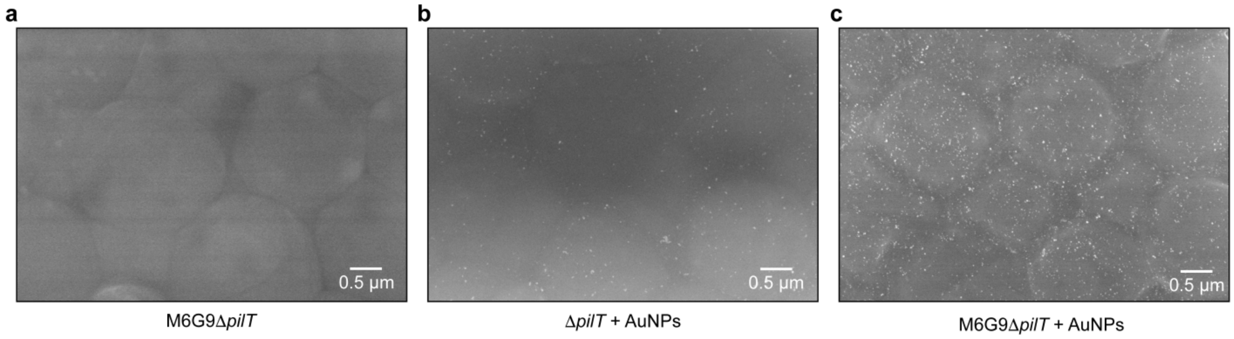

Supplementary Fig. 13. SEM images of *Synechocystis*-AuNPs hybrids. **a**, M6G9Δ*pilT*. **b**, Δ*pilT*+AuNPs. **c**, M6G9Δ*pilT*+AuNPs. The *Synechocystis* cells after incubation with AuNPs were washed with deionized water and the resuspended cells were dropped onto the ITO electrode (10 × 10 mm) for air-dry at room temperature. The surface-dried biofilms were directly subjected for SEM observation. The experiments were repeated three times independently with similar results. Source data are provided as a Source Data file.

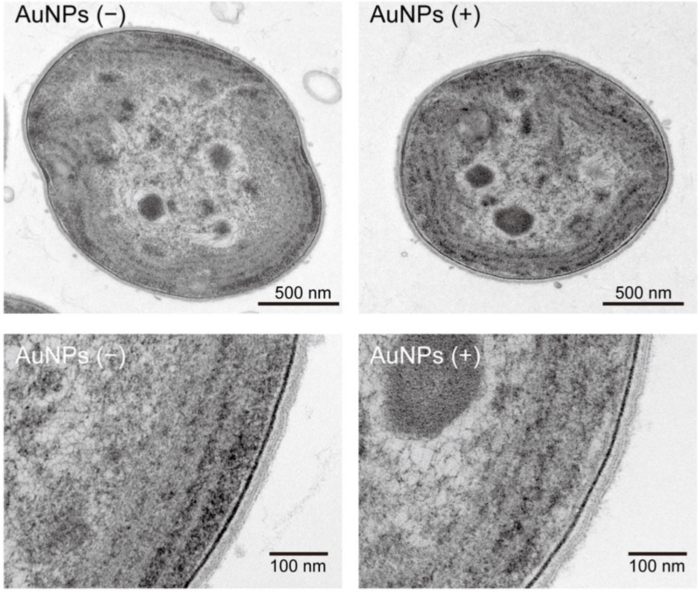

Supplementary Fig. 14. TEM images of cross-sectional M6G9Δ*pilT* cells treated with/without AuNPs. The experiments were repeated three times independently with similar results. Source data are provided as a Source Data file.

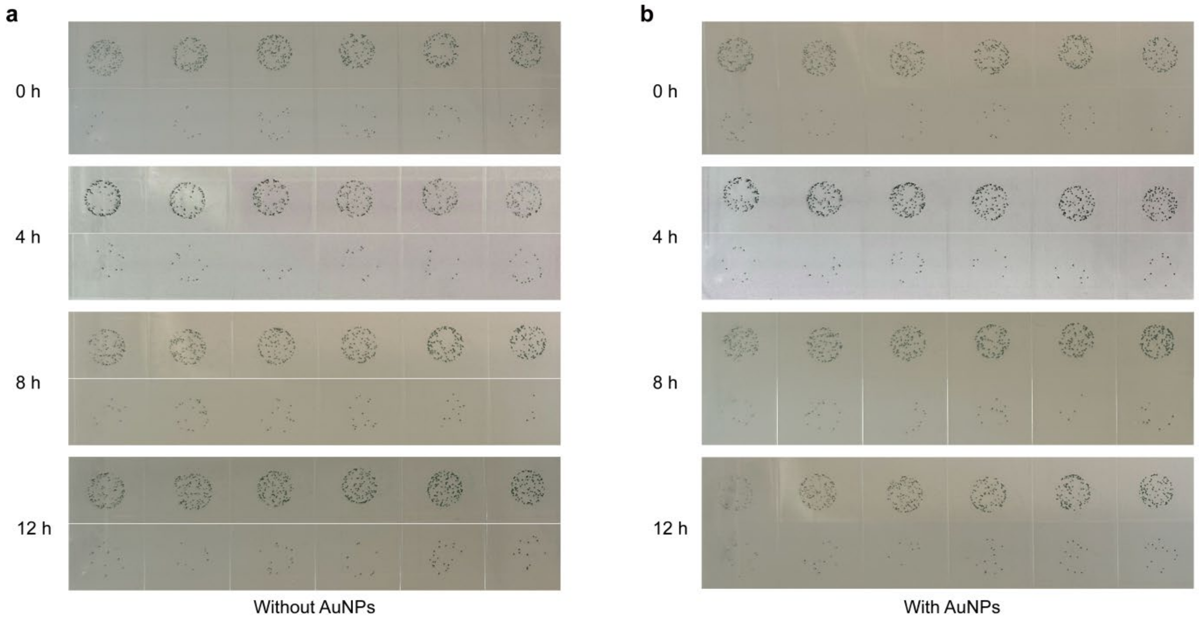

Supplementary Fig. 15. The spot assay of strain M6G9Δ*pilT* treated by AuNPs. a, Without AuNPs treatment. **b**, With AuNPs treatment. The cultures were serially diluted followed by dropping 10 μL of each dilution onto BG11 agar plates. The plates were incubated at 30°C under light for colony formation. Source data are provided as a Source Data file.

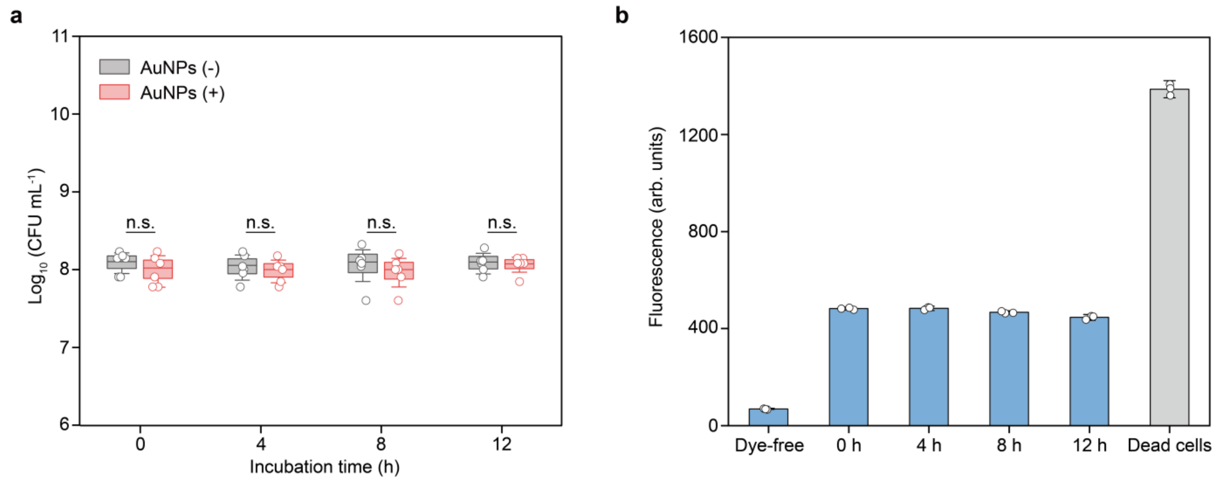

Supplementary Fig. 16. Toxicity assessment of AuNPs for *Synechocystis*. a, Quantitative colony-forming unit (CFU) counts of strain M6G9Δ*pilT* when incubated with AuNPs. Data are presented as mean values ± SD from $n = 6$ independent experiments. **b**, Fluorescence intensity of PI stained *Synechocystis* cells that treated with AuNPs. The dead cells that treated with 70% isopropanol for half an hour was used as the positive control. Data are presented as mean values ± SD from $n = 3$ independent experiments. Source data are provided as a Source Data file.

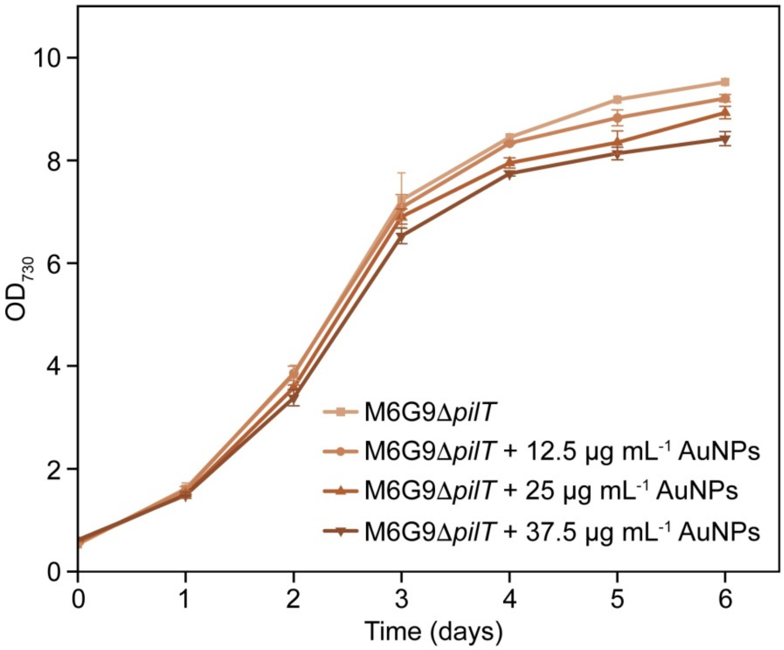

Supplementary Fig. 17. Growth curves of strain M6G9Δ*pilT* in BG11 liquid medium supplemented with different concentrations of AuNPs. OD_{730} represents the cell optical density at 730 nm, which is an indicator of cell growth. Plotted are the means of three independent experiments. Error bars represent standard deviation. Source data are provided as a Source Data file.

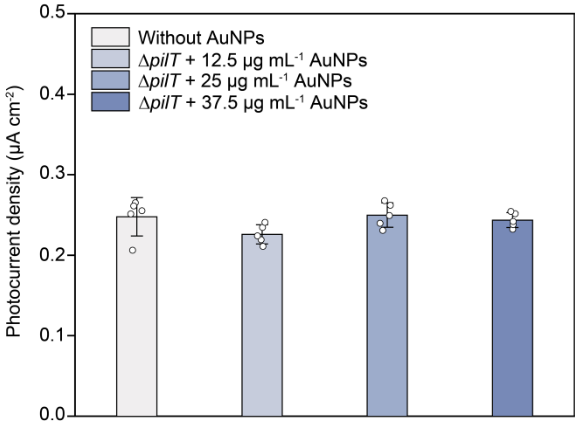

Supplementary Fig. 18. Photocurrent density of strain Δ*pilT* incubated with AuNPs at different concentrations. Data are presented as mean values ± SD from *n* = 5 independent experiments. Source data are provided as a Source Data file.

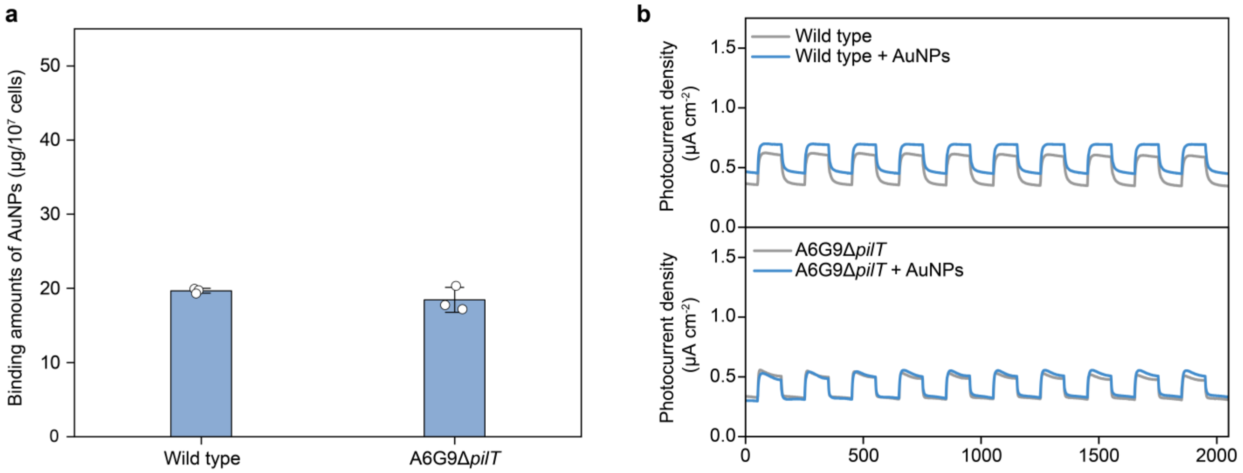

Supplementary Fig. 19. Binding capacity and photocurrent density of other non-targeting control strains including wild type and A6G9Δ*pilT*. a, Binding capacity for AuNPs. Data are presented as mean values ± SD from n = 3 independent experiments. **b**, Photocurrent density in the absence or presence of AuNPs. Source data are provided as a Source Data file.

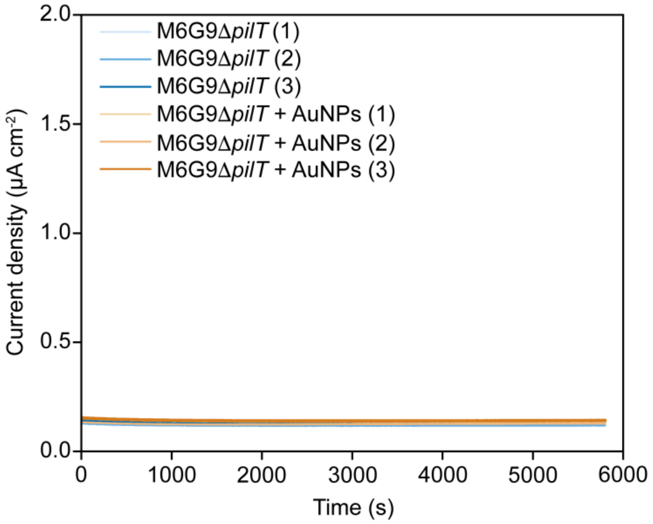

Supplementary Fig. 20. Dark current generated by strain M6G9Δ*pilT* in the absence or presence of AuNPs. The experiments were repeated three times independently. Source data are provided as a Source Data file.

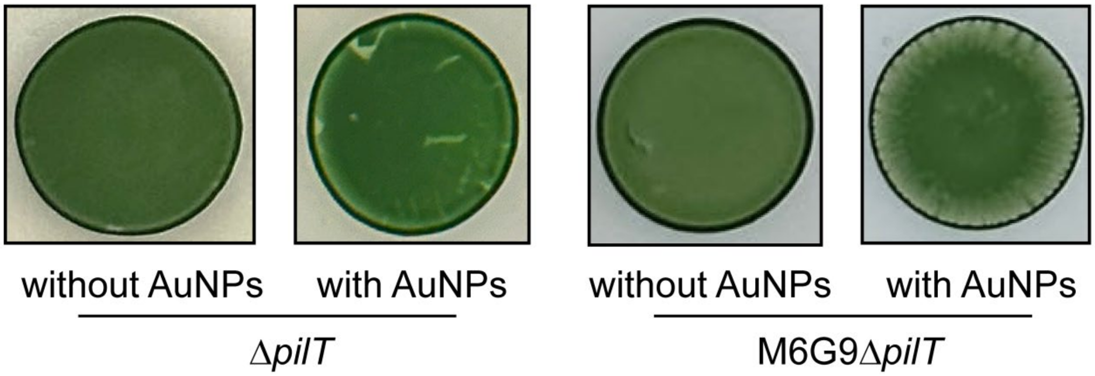

Supplementary Fig. 21. Representative photographs of biofilms formed on ITO electrodes. The experiments were repeated three times independently with similar results. Source data are provided as a Source Data file.

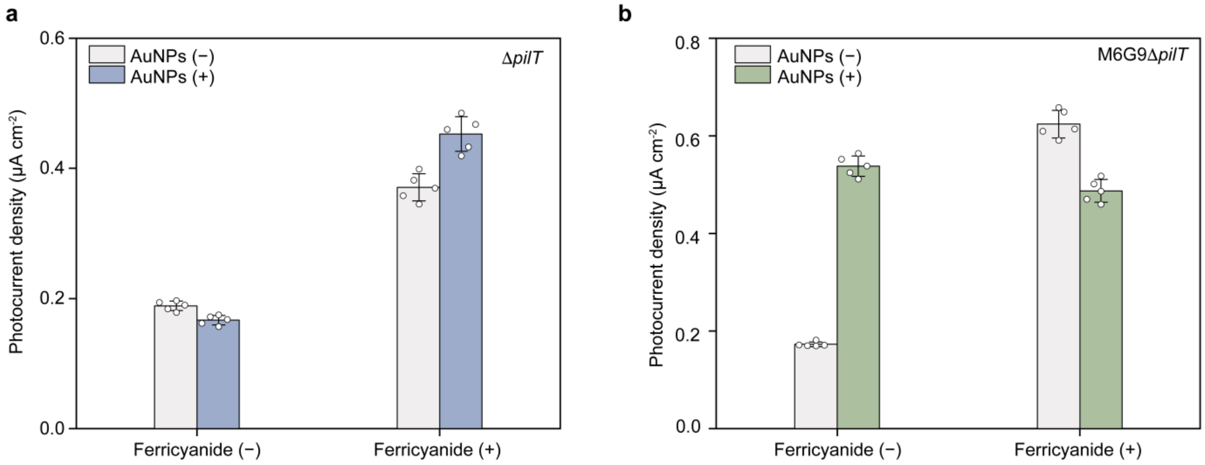

Supplementary Fig. 22. Photocurrent generation of strain Δ*pilT* (a) and M6G9Δ*pilT* (b) under ferricyanide-mediated conditions. Ferricyanide at concentration of 1 mM was added in electrolyte during photocurrent measurement. Data are presented as mean values ± SD from n = 5 independent experiments. Source data are provided as a Source Data file.

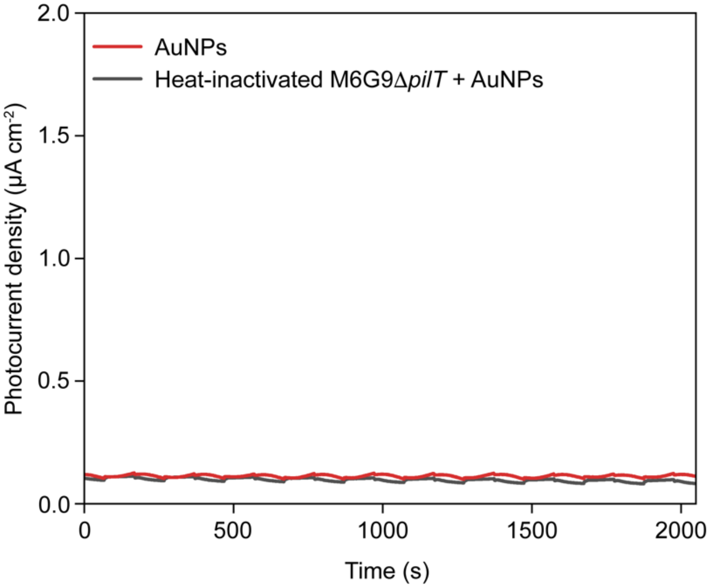

Supplementary Fig. 23. Photocurrent measurement of AuNPs alone or heat-inactivated cells treated with AuNPs. Source data are provided as a Source Data file.

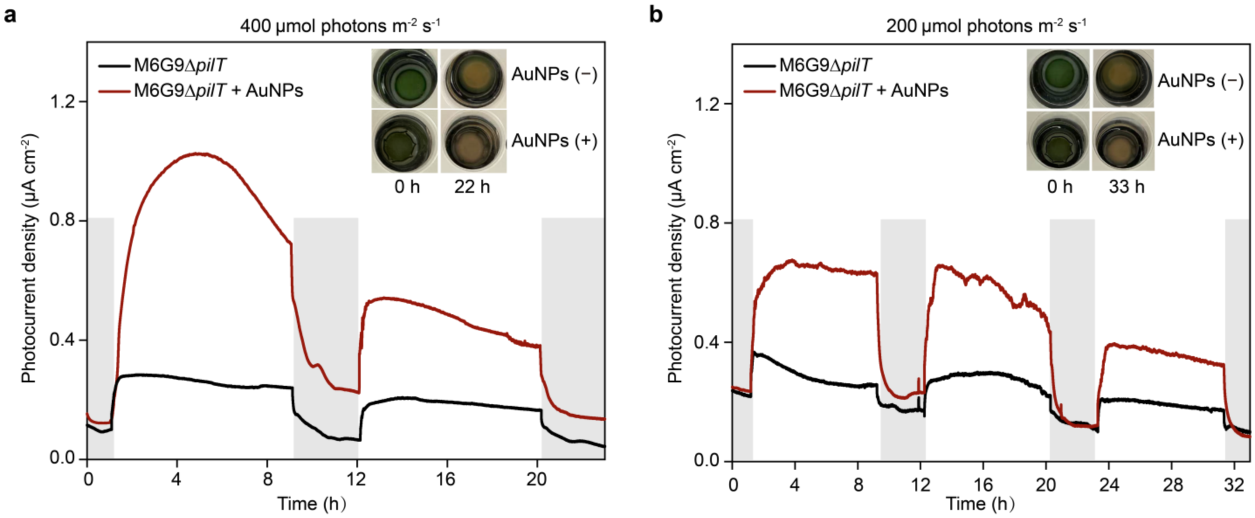

Supplementary Fig. 24. Long-term photocurrent measurements. a, Photocurrent under light intensity of 400 μmol photons m^{-2} s^{-1}. **b**, Photocurrent under light intensity of 200 μmol photons m^{-2} s^{-1}. Chronoamperometry was conducted under continuous illumination for 8 h with 3 h dark intervals. Grey blocks indicate the dark intervals. The insets are photographs showing the cell bleaching of cyanobacteria. Source data are provided as a Source Data file.

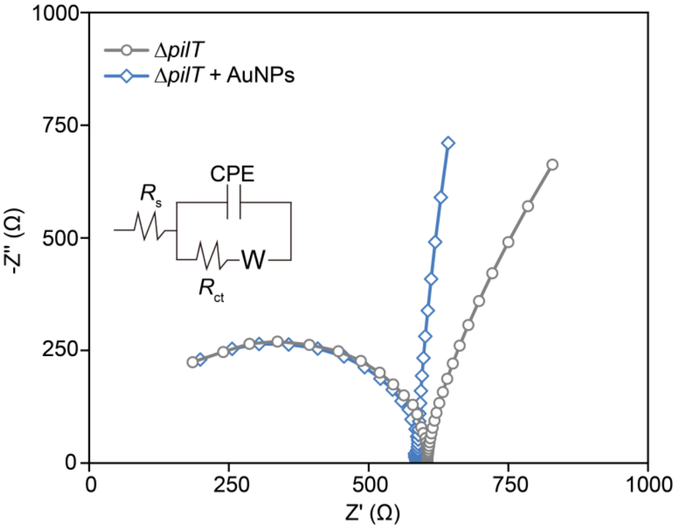

Supplementary Fig. 25. Nyquist plots of the ITO electrode attached by Δ*pilT* cells in the absence or presence of AuNPs. The inset shows the equivalent electrical circuit model used to calculate the resistances. R_s: solution resistance; R_{ct}: charge transfer resistance; CPE: constant phase element; W: Warburg diffusion element. Source data are provided as a Source Data file.

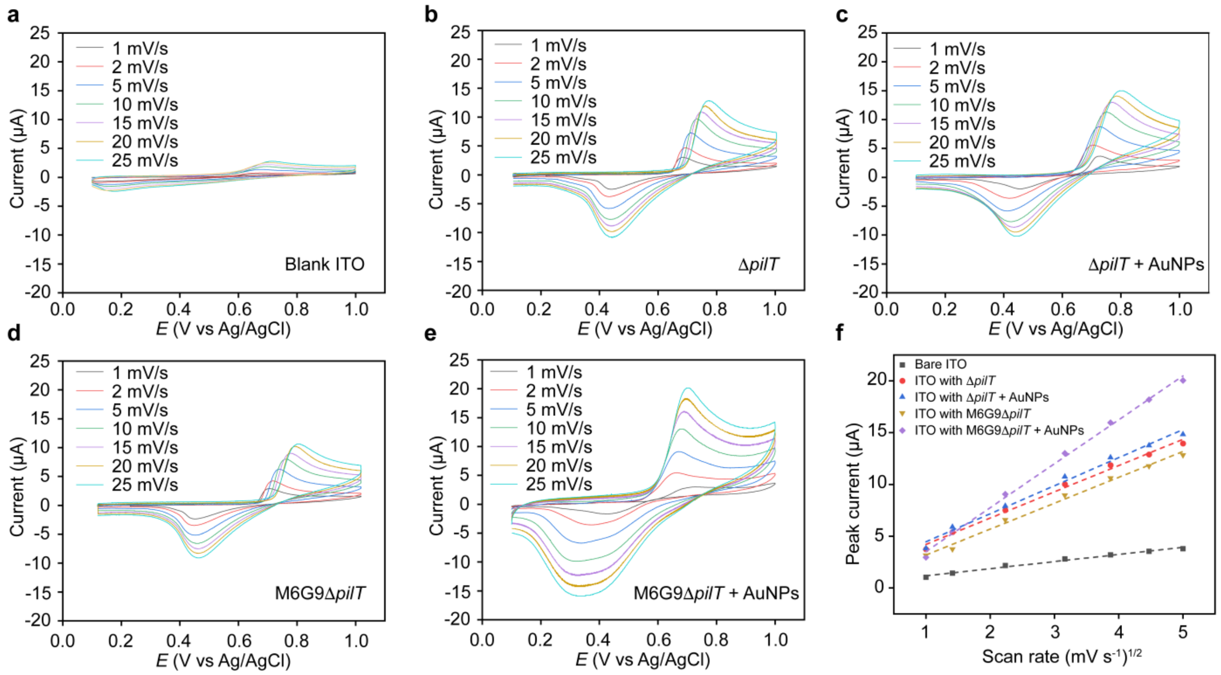

Supplementary Fig. 26. Quantify electrochemically active surface area (ECSA) of biofilm-loaded ITO electrodes. **a**, CV profiles of bare ITO electrode at varying scan rates. **b**, CV profiles of ITO electrode attached by Δ*pilT* cells at varying scan rates. **c**, CV profiles of ITO electrode attached by AuNPs-treated Δ*pilT* cells at varying scan rates. **d**, CV profiles of ITO electrode attached by M6G9Δ*pilT* cells at varying scan rates. **e**, CV profiles of ITO electrode attached by AuNPs-treated M6G9Δ*pilT* cells at varying scan rates. **f**, Peak currents plotted as a function of the square root of the scan rate ($v^{1/2}$). Source data are provided as a Source Data file.

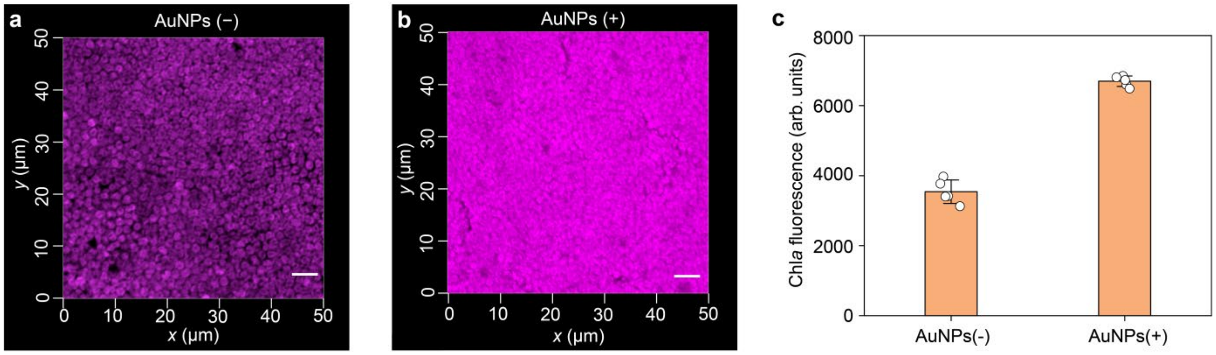

Supplementary Fig. 27. Characterization of the biofilm structure of M6G9Δ*pilT* on ITO electrode. a, CLSM image in the absence of AuNPs. The experiments were repeated three times independently with similar results. **b**, CLSM image in the presence of AuNPs. **c**, Chla fluorescence intensity derived from CLSM images. Data are presented as mean values ± SD from $n = 5$ independent experiments. Source data are provided as a Source Data file.

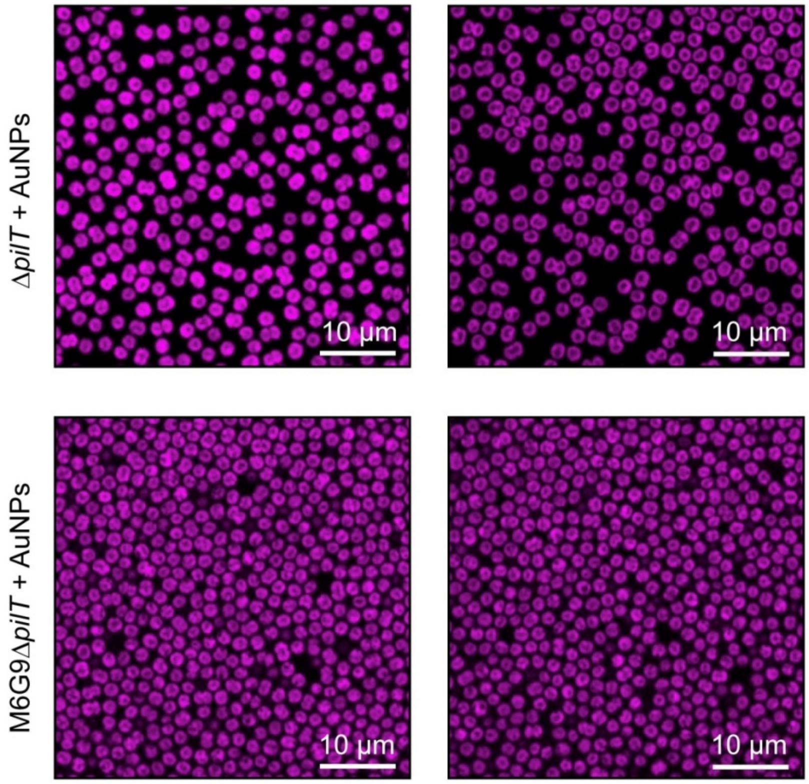

Supplementary Fig. 28. Confocal microscopy images of the biofilms that tightly attached onto the ITO electrode. The top row represents strain Δ*pilT*, and the bottom row represents strain M6G9Δ*pilT*. Purple autofluorescence indicates photosynthetic pigments. The strain M6G9Δ*pilT* cells exhibited more cells that tightly attached onto the electrode when compared to strain Δ*pilT*. The experiments were repeated three times independently with similar results. Source data are provided as a Source Data file.

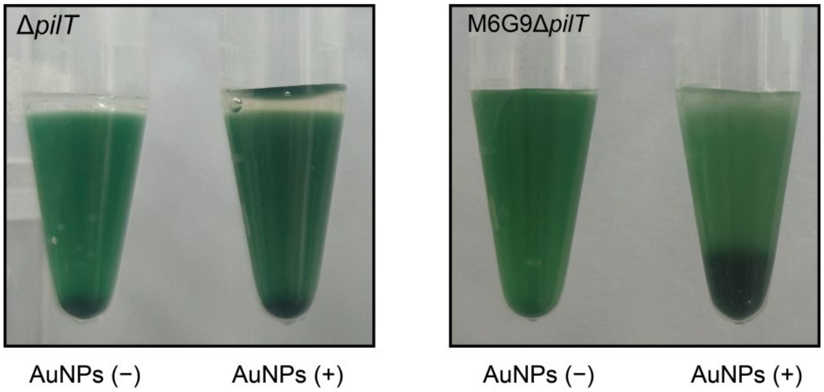

Supplementary Fig. 29. The photographs of aggregation phenomenon of engineered strains incubated with AuNPs. Aggregation was occurred when M6G9Δ*pilT* cells were incubated with AuNPs. The experiments were repeated three times independently with similar results. Source data are provided as a Source Data file.

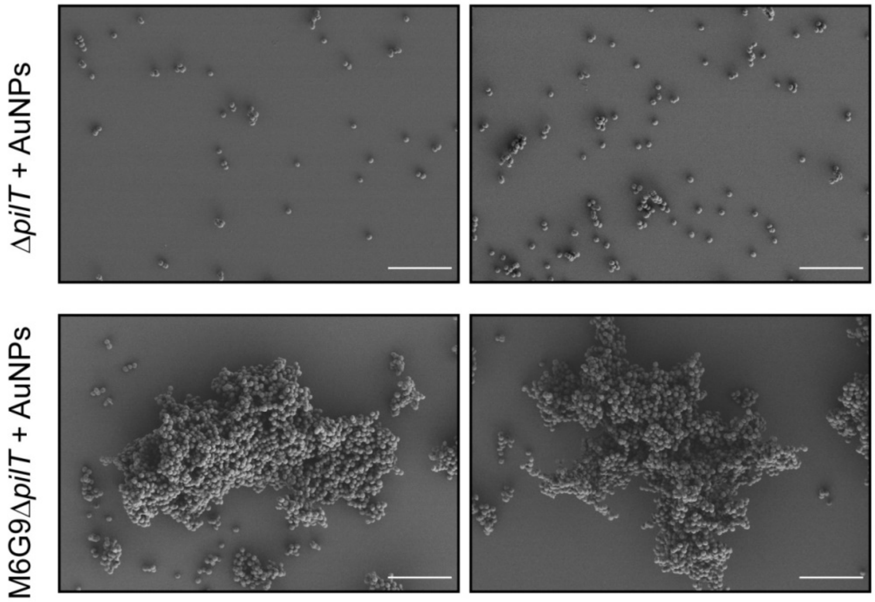

Supplementary Fig. 30. SEM images of *Synechocystis*-AuNPs hybrids. Two individual images of strain Δ*pilT* (top) and strain M6G9Δ*pilT* (bottom) were selected for comparison. Once treatment with AuNPs, M6G9Δ*pilT* cells formed compact clusters, whereas Δ*pilT* cells remained scattered. Scale bar: 20 μm. The experiments were repeated three times independently with similar results. Source data are provided as a Source Data file.

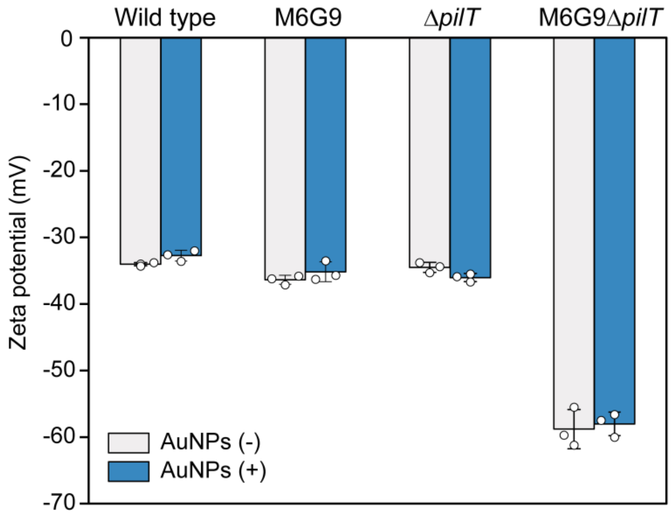

Supplementary Fig. 31. Zeta potentials of different *Synechocystis* strains before and after treatment with AuNPs. The treatment with AuNPs did not change the zeta potentials of wild-type and engineered *Synechocystis* strains. Data are presented as mean values ± SD from *n* = 3 independent experiments. Source data are provided as a Source Data file.

Supplementary Tables

Supplementary Table 1. The sequences of gold binding peptides (GBPs) used in this study.

ID	Name	Sequence (AA)
GBP_1	M6G9	MMMGGGMGGGMGGGM
GBP_2	GBP1	MHGKTQATSGTTQS
GBP_3	P_3Au12	LKAHLPPSRLPS
GBP_4	A13	IIMNLSGNPAPTDHS
GBP_5	P8#9	VSGSSPDS
GBP_6	P8#17	GLDGSAVD
GBP_7	I24	SQHVRRCVRLLALDL
NC	Negative control	GTSGQ (linker only)
M6G9 mutant_1	M3A3G9	MMMGGGAGGGAGGGA
M6G9 mutant_2	A3M3G9	AAAGGGMGGGMGGGM
M6G9 mutant_3	A6G9	AAAGGGAGGGAGGGA

Supplementary Table 2. The strains used in this study.

Strains	Descriptions	Sources
Escherichia coli		
E. coli DH5 α	Host for plasmid construction	TransGen
E. coli BL21(DE3)	Host for surface display	TransGen
Synechocystis sp. PCC 6803		
Wild type	Main host in this study	Lab storage
ΔpilA	*pilA1* (*sll1694*) disruption, replaced by Cm^R insertion	This study
ΔpilT	*pilT* (*slr0161*) disruption, replaced by Gm^R insertion	This study
M6G9	Wild type with *pilA-M6G9* (P_{cpc560}) integrated at NSI site	This study
M6G9Δ*pilT*	Δ*pilT* with *pilA-M6G9* (P_{cpc560}) integrated at NSI site	This study
PilA-M6G9	Wild type with *pilA-M6G9* (P_{rbcL}) integrated at *pilA* site	This study
P_{rbcL}-M6G9	Wild type with *pilA-M6G9* (P_{rbcL}) integrated at NSI site	This study

Supplementary Table 3. The plasmids used in this study.

Plasmids	Descriptions	Sources
pET30a	ColE1, T7 promoter, Kan^R	Genscript
pET30a-GFP	pET30a vector for expression OMP-GFP in *E. coli*	This study
pET30a-GBP	pET30a vector for expression eCPX-GBP in *E. coli*	This study
pUC57	f1 ori, lac promoter, Amp^R	Genscript
NSI-P_{cpc560}-PilA-M6G9	pUC57::P_{cpc560}-NSI_up-PilA-M6G9-T_{rbcL}-NSI_down, NSI targeting, Amp^R $Spec^R$	This study
NSI-P_{rbcL}-PilA-M6G9	pUC57::P_{rbcL}-NSI_up-PilA-M6G9-T_{rbcL}-NSI_down, NSI targeting, Amp^R $Spec^R$	This study
P_{cpc560}-PilA-M6G9	pUC57::P_{rbcL}-*pilA1*_up-PilA-M6G9-T_{rbcL}-*pilA1*_down, *pilA1* targeting, Amp^R $Spec^R$	This study
pUC-Δ*pilA*	Suicide vector for *pilA1* (*sll1694*) knockout, Amp^R Cm^R	This study
pUC-Δ*pilT*	Suicide vector for *pilT* (*slr0161*) knockout, Amp^R Gm^R	This study

OMP: outer membrane protein, GBP: gold binding peptide

Supplementary Table 4. DNA sequences of the primers used in this study.

Primers	Sequences (5'-3')
pilA1 up-F	TTGTAAAACGACGGCCAGTGAATTCATGGCATTATTCGGGGTAATCTCCA
pilA1 up-R	TGTAATCAGCACAGTTCATTATCAACGATTGTCTTCTTCCTTCTGTAGGG
*pilA1*down-F	AAGCGGATGAATGGCAGAAATTCGATGACCCTATTATGTTTTGAGTGGTG
pilA1 down-R	CTATGACCATGATTACGCCAAGCTTCAAAAATAGAAGTGCTAGTTTCGAG
pilT up-F	TTGTAAAACGACGGCCAGTGAATTCTCTTCGGCTAATAGTCGGAA
pilT up-R	GTGAGCCAGAGTTTCAGACTTTAATGCTCCTATAAGTTCT
pilT down-F	TCGTTCCACTGAGCGTCACATACGGAAATATACCGATTAAC
pilT down-R	TATGACCATGATTACGCCAAGCTTGGCACAGTATTACAAACAATTTCCAA
NS1 up-F	CAGTGAATTCGCAGACTTACGTTTTCTC
NS1 up-R	GAATGAGCTCCTTGGCTCTTTTTCCTGG
NS1 down-F	GAAATTCGATTCACTCTATGGTGATTATGAG
NS1 down-R	CGCCAAGCTTGCCGTGGATAAATCAAAC
P_{cpc560}-F	GACACCACAACCCAGGAAAAAGAGCCAAGACCTGTAGAGAAGAGTCC
P_{cpc560}-R	GAATTTAAAATTACTAGCCATTGAATTAATCTCCTACTTGACTTTA
P_{rbcL}-F	ACCCAGGAAAAAGAGCCAAGGGGCTTCAATAAATGGTTCCGA
P_{rbcL}-R	GTTTGAATTTAAAATTACTAGCCATGTCGTCTCTCCCTAGAGATATGTCA
PilA-M6G9-F	ATGGCTAGTAATTTTAAATTCAAAC
PilA-M6G9-R	TACATACCGCCTCCCATGCCTCCAC
Cm-F	CCCTACAGAAGGAAGAAGACAATCGTTGATAATGAACTGTGCTGATTACA
Cm-R	CACCACTCAAAACATAATAGGGTCATCGAATTTCTGCCATTCATCCGCTT
Gm-F	CTTATAGGAGCATTAAAGTCTGAAACTCTGGCTCACCGAC
Gm-R	ATCGGTATATTTCCGTATGTGACGCTCAGTGGAACGAAAAC
Spec-F	GTGGGGAATTGTCAATTGTCAATTATCGAGTTCATGTGCAGCTCCATAAG
Spec-R	CATAATCACCATAGAGTGATTATTTGCCGACTACCTTGGT
T_{rbcL}-F	TATGGGTGGAGGCATGGGAGGCGGTATGTAGGTTACAGTTTTGGCAATTACT
T_{rbcL}-R	CTTATGGAGCTGCACATGAACTCGATAATTGACAATTGACAATTCCCCAC
PilA-F	CAGCTAGAAGGGTTCCGACC
PilA -R	TCATTGCTCCCAACACTCCC
PilT-F	TGGCTGTGGGTTCAGAACTT
PilT-R	TGATTAGGGGGATTGCACCG
NS1-F	CAGAAAGGCTAGGGTTGGGG
NS1-R	CGTGTGCTTTTTCCAGAGGC

Supplementary Table 5. Comparison of Au material demand in different studies.

Photosynthetic microorganisms	Power output improvement vs. untreated cells (~fold)	AuNPs conc. in solutions (mmol L^{-1})	Number of cells used	Au demand per cell (mol)	Treatment time (h)	References
Chlamydomonas reinhardtii	1.74	0.00635	2.0×10^{6}	6.4×10^{-15}	3	46
Synechocystis sp. PCC 6803	33.6	1	5.4×10^{7}	1.8×10^{-14}	16	48
Synechocystis sp. PCC 6803	4.3	0.127	2.7×10^{8}	9.4×10^{-17}	2	This work
