# Supplementary material for: Engineered conductive pili enable high-efficiency photosynthetic electron extraction in biophotovoltaics
Source: Nat Commun. 2026 Apr 25;17:5724. doi: 10.1038/s41467-026-72407-7 (PMC13323719; doi:10.1038/s41467-026-72407-7)
Supplement: Supplementary file 2 — Description of Additional Supplementary Files [file 41467_2026_72407_MOESM2_ESM.pdf]

Description of Additional Supplementary Files

Title: Supplementary Data 1

Description: The performance of previously reported biophotovoltaics (BPV) systems.
