# Supplementary material for: Engineered conductive pili enable high-efficiency photosynthetic electron extraction in biophotovoltaics
Source: Nat Commun. 2026 Apr 25;17:5724. doi: 10.1038/s41467-026-72407-7 (PMC13323719; doi:10.1038/s41467-026-72407-7)
Supplement: Supplementary file 4 — Reporting Summary [file 41467_2026_72407_MOESM4_ESM.pdf]

nature portfolio

Corresponding author(s): Yanping Zhang, Yin Li, Huawei Zhu

Last updated by author(s): Mar 11, 2026

Reporting Summary

Nature Portfolio wishes to improve the reproducibility of the work that we publish. This form provides structure for consistency and transparency in reporting. For further information on Nature Portfolio policies, see our Editorial Policies and the Editorial Policy Checklist.

Statistics

For all statistical analyses, confirm that the following items are present in the figure legend, table legend, main text, or Methods section.

n/a	Confirmed	
☐	☒	The exact sample size (*n*) for each experimental group/condition, given as a discrete number and unit of measurement
☐	☒	A statement on whether measurements were taken from distinct samples or whether the same sample was measured repeatedly
☐	☒	The statistical test(s) used AND whether they are one- or two-sided<br>*Only common tests should be described solely by name; describe more complex techniques in the Methods section.*
☒	☐	A description of all covariates tested
☒	☐	A description of any assumptions or corrections, such as tests of normality and adjustment for multiple comparisons
☐	☒	A full description of the statistical parameters including central tendency (e.g. means) or other basic estimates (e.g. regression coefficient) AND variation (e.g. standard deviation) or associated estimates of uncertainty (e.g. confidence intervals)
☒	☐	For null hypothesis testing, the test statistic (e.g. *F*, *t*, *r*) with confidence intervals, effect sizes, degrees of freedom and *P* value noted<br>*Give P values as exact values whenever suitable.*
☒	☐	For Bayesian analysis, information on the choice of priors and Markov chain Monte Carlo settings
☒	☐	For hierarchical and complex designs, identification of the appropriate level for tests and full reporting of outcomes
☒	☐	Estimates of effect sizes (e.g. Cohen's *d*, Pearson's *r*), indicating how they were calculated

Our web collection on statistics for biologists contains articles on many of the points above.

Software and code

Policy information about availability of computer code

Data collection	A CHI1030C potentiostat and associated electrohemical software chi1030c (version 16.6.0.0) were used for electrochemical data collection. A CHI660E electrochemical workstation was used for EIS analysis. The Nyquist plots were fitted to an equivalent circuit model using the software ZSimpWin (version 3.60). A TU-1900 UV–VIS spectrophotometer (Persee, Beijing, China) was used to measure the absorbance.
Data analysis	Microsoft Excel 2010, Origin 2024, GraphPad Prism 7 and Adobe Illustrator 2020 were used for data analysis and graphing.

For manuscripts utilizing custom algorithms or software that are central to the research but not yet described in published literature, software must be made available to editors and reviewers. We strongly encourage code deposition in a community repository (e.g. GitHub). See the Nature Portfolio guidelines for submitting code & software for further information.

Data

Policy information about availability of data

All manuscripts must include a data availability statement. This statement should provide the following information, where applicable:

- Accession codes, unique identifiers, or web links for publicly available datasets
- A description of any restrictions on data availability
- For clinical datasets or third party data, please ensure that the statement adheres to our policy

All data supporting the findings of this study are available within the article and its supplementary files. Any additional requests for information can be directed to, and will be fulfilled by, the corresponding authors. Source data are provided with this paper.

April 2023

Research involving human participants, their data, or biological material

Policy information about studies with human participants or human data. See also policy information about sex, gender (identity/presentation), and sexual orientation and race, ethnicity and racism.

Reporting on sex and gender	Human participants were not involved in this study.
Reporting on race, ethnicity, or other socially relevant groupings	Human participants were not involved in this study.
Population characteristics	Human participants were not involved in this study.
Recruitment	Human participants were not involved in this study.
Ethics oversight	Human participants were not involved in this study.

Note that full information on the approval of the study protocol must also be provided in the manuscript.

Field-specific reporting

Please select the one below that is the best fit for your research. If you are not sure, read the appropriate sections before making your selection.

☒ Life sciences ☐ Behavioural & social sciences ☐ Ecological, evolutionary & environmental sciences

For a reference copy of the document with all sections, see nature.com/documents/nr-reporting-summary-flat.pdf

Life sciences study design

All studies must disclose on these points even when the disclosure is negative.

Sample size	The sample size of n = 3 biological replicates was chosen according to the standards of the field (at least three independent biological replicates for statistical analysis) to ensure reproducibility while minimizing cost.
Data exclusions	No data were excluded from analysis.
Replication	All experiments were independently repeated at least three times with good reproducibility.
Randomization	The electrochemical devices and cultivation flasks used in all experiments were randomly placed in illumination/dark incubators.
Blinding	Blinding was not performed because the data collection was recorded by well-controlled instruments. In addition, the data are quantitative and appropriate control experiments were included, so the subjective judgment or interpretation was not involved during data analysis.

Reporting for specific materials, systems and methods

We require information from authors about some types of materials, experimental systems and methods used in many studies. Here, indicate whether each material, system or method listed is relevant to your study. If you are not sure if a list item applies to your research, read the appropriate section before selecting a response.

Materials & experimental systems

n/a	Involved in the study	
☐	☒	Antibodies
☒	☐	Eukaryotic cell lines
☒	☐	Palaeontology and archaeology
☒	☐	Animals and other organisms
☒	☐	Clinical data
☒	☐	Dual use research of concern
☒	☐	Plants

Methods

n/a	Involved in the study	
☒	☐	ChIP-seq
☒	☐	Flow cytometry
☒	☐	MRI-based neuroimaging

Antibodies

Antibodies used	AF647-conjugated anti-GFP antibody (AC1432, Beyotime, 1:20 dilution), a primary rabbit polyclonal antibody against PilA1 (Abclonal, 1:3000 dilution) and a secondary Goat-anti Rabbit-HRP antibody (BE0103, Easybio, 1:3000 dilution) were used in this study.

Validation	The above listed antibodies were used according to the manufactures' instructions.

Plants

Seed stocks	N/A
Novel plant genotypes	N/A
Authentication	N/A
