# Supplementary material for: Engineered conductive pili enable high-efficiency photosynthetic electron extraction in biophotovoltaics
Source: Nat Commun. 2026 Apr 25;17:5724. doi: 10.1038/s41467-026-72407-7 (PMC13323719; doi:10.1038/s41467-026-72407-7)
Supplement: Supplementary file 5 — Transparent Peer Review file [file 41467_2026_72407_MOESM5_ESM.pdf]

nature portfolio

Peer Review File

Engineered conductive pili enable high-efficiency photosynthetic electron extraction in biophotovoltaics

Corresponding Author: Professor Huawei Zhu

This file contains all reviewer reports in order by version, followed by all author rebuttals in order by version.

Version 0:

Reviewer comments:

Reviewer #1

(Remarks to the Author)
The authors present an innovative synthetic-biology–guided strategy to direct gold nanoparticles (AuNPs) onto type IV pili of Synechocystis sp. PCC 6803 by fusing a gold-binding peptide to PilA1. This targeted assembly creates a conductive nano-bio interface on the cell envelope, lowers interfacial charge-transfer resistance by about 23%, and increases mediator-free photocurrent on planar ITO by approximately 4-fold, while using substantially less gold than non-targeted approaches. The work addresses a key bottleneck in biophotovoltaics—inefficient extracellular electron transfer (EET)—and offers a conceptually appealing, material-efficient route to engineer bio-electronic interfaces with spatial precision. Despite these advances, several issues should be addressed before publication.

1. In Figure 4d, the TEM image shows AuNPs distributed along pili outside the cell. The interparticle distances (>50 nm) appear too large to support an effective electrical pathway. Additional evidence that pili-localized AuNPs form a percolating, conductive network interfacing with the electrode would substantially strengthen the mechanistic claims.

2. For photocurrent measurements, “pre-incubated mixture of Synechocystis cells and AuNPs were dropped onto an ITO conductive glass and allowed to air-dry.” However, non-adherent cells may gradually detach, as noted in the biofilm formation assay. Please discuss the “loading” effects.

3. The manuscript attributes photocurrent gains to enhanced cell–electrode charge transfer and increased intercellular interactions after AuNP functionalization. Biofilm formation likely also contribute. Please characterize biofilm structure (e.g., thickness, density, porosity) and discuss how these features affect EIS parameters.

4. For strain M6G9ΔpilT treated with AuNPs, Rct decreases by ~20%, and biofilm formation is only slightly improved (Fig. 7c). Yet the photocurrent density increases by ~4.3-fold (Fig. 5b). Please clarify the primary mechanism(s) underlying this disproportionate photocurrent enhancement.

5. Include long-term chronoamperometry under continuous illumination (hours to days) to assess stability beyond light/dark transients. If performance decays, discuss whether the cause is physiological changes, nanoparticle detachment/aggregation, or photodamage.

6. Zeta potential measurements in deionized water do not reflect the ionic strength of BG11. Please report zeta potential in BG11 (or a buffer with matched ionic strength) for physiologically relevant interpretation.

7.Textual edits and typos
“an cutting-edge” → “a cutting-edge”
“an photocurrent” → “a photocurrent”
“can functioned as” → “can function as”

Reviewer #2

(Remarks to the Author)
In the manuscript of Wang et al., the authors address a key challenge in biophotovoltaics: the efficient extraction of electrons

from photosynthetic microorganisms. They present a targeted biohybrid strategy using genetically encoded gold-binding peptides to direct the assembly of gold nanoparticles (AuNPs) onto the type IV pili of Synechocystis sp. PCC 6803. This creates a defined conductive interface that enhances electron transfer from the cells to external electrodes. Their approach claims a reduction in the interfacial resistance by 23% and achieves a four-fold increase in photocurrent density, while requiring significantly less gold compared to non-targeted methods. The study also claims to identify key methionine residues responsible for AuNP binding and demonstrates the potential for functional transfer of AuNPs between cells, suggesting long-term system stability. Overall, the work offers a generalizable framework for engineering conductive interfaces on living cells for applications in biophotovoltaics and other biohybrid technologies.

The manuscript is clearly written, methodologically sound, and visually supported by high-quality figures (notably, Figure 1, which is exceptionally well designed and informative).
That said, I have one main point of concern that I believe should be addressed before the manuscript is considered for publication.

Major Comment – Specificity of Enhanced Electron Flow to Photocurrent
While the enhanced photocurrent observed in the M6G9ΔpilT + AuNPs strain compared to M6G9ΔpilT alone is compelling, I find it puzzling that the engineered conductive pili appear to facilitate only photosynthetic electron extraction, with no enhancement in dark current. This outcome is unexpected, particularly given that in Synechocystis, the photosynthetic and respiratory electron transport chains share common electron carriers and are known to exchange electrons.
The authors should provide a robust mechanistic explanation or at least a thoughtful discussion on why this selective enhancement occurs.
Without such clarification, the interpretation of the photocurrent enhancement remains incomplete.

Additional Comments
1. Clarification on Benchmarking of BPV Performance in the Introduction
The manuscript states:
“It was estimated that the theoretically maximum photocurrent density of BPV is 340 μA cm^{-2}, a value comparable to that of commercial photovoltaic (PV) technology [7].”
This appears to be a misinterpretation of reference [7] (McCormick et al.). That paper refers to an “achievable” current and power output range, not a theoretical maximum. Furthermore:
• The estimate includes both light and dark current components.
• McCormick et al. reported a range of values (340–2460 μA cm^{-2}) based on lighting conditions, not a single value.
• The claim of comparability to commercial PVs is inconsistent with McCormick et al.’s statement:
“... the gap with recently built solar power stations is substantially larger (ca. 70-fold).”
I encourage the authors to revise this section for accuracy and consistency with the original source.

2. Durability of AuNP-Tagged Synechocystis
A key factor for the practical application of this biohybrid system is its long-term stability. I would welcome the inclusion of a longer-term chronoamperometry dataset (e.g., over 10 days), to assess how the photocurrent performance of AuNP-tagged cells evolves over time. While not essential for acceptance, such data would significantly strengthen the manuscript.

3. Inheritance of AuNPs in Regenerating Cells
Following the previous point, I am curious about the behavior of AuNPs during Synechocystis cell division. If the cells regenerate, do the nanoparticles remain associated with daughter cells, or is re-functionalization necessary? Some discussion or preliminary observation in this direction would be appreciated, as it could have important implications for system scalability and long-term use.

Reviewer #3

(Remarks to the Author)
To address the low photosynthetic electron extraction efficiency in bio-photovoltaics (BPV), this study combined synthetic biology with targeted nano-assembly to conduct the following research: They screened the high-affinity gold-binding peptide M6G9, fused its gene to the PilA1 subunit of Synechococcus phycobacterium type IV pili, and simultaneously knocked out the pili retraction gene to construct a hyper-piliated strain; Leveraging M6G9's specific binding to gold nanoparticles (AuNPs), the study achieved ordered assembly of AuNPs on the pili, forming an extracellular conductive network. Ultimately, this approach increased photocurrent density by 4.3-fold, reduced interfacial electron transfer resistance by 23%, and decreased gold consumption by two orders of magnitude while enabling recyclability and maintaining excellent biocompatibility. After carefully reviewing and analyzing the manuscript, we believe the following improvements are still needed:

1. In the targeting strategy, gold forms Au-S bonds with methionine in M6G9. Compared to non-targeting physical adsorption, this chemical interaction is likely to produce distinct characteristic peaks. Therefore, X-ray photoelectron spectroscopy (XPS) can be employed to analyze the chemical state of Au, allowing for the differentiation of the interfacial formation mechanisms between the two strategies at the chemical level and thereby providing a more comprehensive comparative analysis.
2. The exclusive reliance on "ΔpilT+AuNPs (physical adsorption)" as the non-targeting control limits the scope of comparison. To fully substantiate the superiority of the proposed targeting strategy in terms of interfacial consistency and material utilization, it is advisable to include at least two additional non-targeting control groups. A unified comparison of key parameters—such as Au consumption, photocurrent density, and interfacial homogeneity (quantified by TEM analysis)—

would provide a more robust evaluation, highlighting the innovation of this study through a systematic target versus non-target comparison.
3. For the 50-65% recovery rate of AuNPs, optimization methods should be proposed to improve the recovery rate.
4. The current approach to AuNP size optimization is limited, as only three sizes were tested. Expanding the range to include smaller (e.g., 5 nm) and larger (e.g., 80/100 nm) particles would help identify the optimal size interval and determine whether performance peaks at 10 nm or is sustained across a broader range (e.g., 5–10 nm). Furthermore, incorporating molecular docking simulations to study M6G9 interactions with different AuNP sizes, along with quantitative TEM analysis of AuNP assembly density on pili, would provide critical evidence for the "binding pocket matching" hypothesis and substantially improve the mechanistic discussion.
5. A thorough review of the article's writing format is required. For instance, "25 mg ml-1" in line 241 should be revised to "25 mg mL-1", and the same applies to instances like "30 μg ml-1, 50 μg ml-1, 20 μg ml-1" in line 562. Additionally, abbreviations in the text should be clearly defined upon first use, such as supplementing "photosynthetic electron transport chains" with "(PETCs)" in the abstract.

Reviewer #4

(Remarks to the Author)
Biophotovoltaic systems are promising and sustainable approaches for directly converting sunlight into electrical energy with the help of living photosynthetic microorganisms. Drawing upon photosynthetic electron extraction, especially from engineered bacterial platforms, has gained more recent interest because of its potential to enhance electron transfer efficiencies that are not achievable in naturally occurring strains. In this work, a targeted nano-bioengineering strategy for enhanced photosynthetic electron extraction has been reported, whereby Synechocystis cells have been engineered to assemble gold nanoparticles onto their type IV pili, yielding a conductive interface that significantly lowers electron-transfer resistance and boosts photocurrent output using minimal amounts of gold. The authors have established a well-structured series of experiments that encompass most of the key components necessary for analyzing photosynthetically derived extracellular electron transfer. Generally, their experimental approach is sound and incorporates multiple complementary aspects. The following are my concerns and suggestions: The authors demonstrate, using CLSM, that the expression of the eCPX(N)-Spacer construct in E. coli results in peripheral green fluorescence, which they use to indicate preferential localization near the cell envelope. While CLSM is an appropriate indication, it cannot be used alone to establish that a GFP-fusion protein is surface-exposed. Complementary surface-accessibility assays must be included to support such a claim. Methods such as protease shaving of intact cells (e.g., treatment with proteinase K or trypsin), non-permeable biotinization, or antibody labeling, combined with flow cytometric analysis, would directly test whether the fluorescent domain is accessible on the outside of the bacterial envelope. Furthermore, immunogold labeling followed by electron microscopy would yield ultrastructural confirmation of surface localization. The inclusion of one or more of these orthogonal approaches would substantially strengthen proof of true surface display. The terminology and explanation of zeta potential require revision for scientific accuracy. Line 234: the phrase “a decrease in the zeta potential” is misleading, because a reduction in the magnitude of the (negative) zeta potential would be expected to diminish rather than enhance suspension stability. Similarly, the expression at line 518, “reduced the negative potential,” is scientifically incorrect as written. The manuscript wording at present may confuse readers, as although the numerical value becomes more negative, the magnitude of the zeta potential is actually increasing; it is this higher absolute value that strengthens electrostatic repulsion and improves dispersion. I suggest that both be reworded to clearly distinguish between numerical decreases - i.e., more negative values - and increases in the magnitude of the zeta potential, to avoid misunderstandings and ensure the proper description of the relationship between surface charge and suspension stability. In the spot assay, the treatment with AuNPs at 12 h gives fewer colonies compared to the other groups, especially when compared to the “without AuNPs at 12 h” condition. This is not sufficient to support the authors’ conclusion that nanoparticle enrichment does not affect growth. I recommend that the authors provide quantitative CFU counts from biological replicates at changing time points, accompanied by appropriate statistical analyses. NP-only control spots on agar should also be included to exclude physical or optical artefacts. Alternatively, an orthogonal viability assay-such as CFU enumeration or live/dead staining at 12 h-would strengthen the interpretation. If the viability gets affected, the authors should report the effect size and discuss whether the decrease reflects a transient growth delay or nanoparticle-induced toxicity, and adjust their conclusions accordingly. In this work, the authors introduce a mediator-free biophotovoltaic system that utilizes targeted AuNP enrichment and controllable nanoparticle assembly to engineer a hybrid conductive layer on the photosynthetic cell envelope. This approach is taken in order to enhance extracellular electron export and offer a potentially universal strategy for intensifying extracellular energy conversion. Discussion: Well elaborated; the authors interpret their results in detail; the advantages of their design, the shortcomings of current approaches, and the way to cope with these are clearly outlined. Comparison with previous literature is appropriate and relevant, enabling the reader to understand the technological advancements that have been made. In particular, the choice of type IV pili as a biological scaffold to build a living biophotovoltaic interface is well motivated, hence justifying the causality between pilus engineering and improved electron extraction.

Version 1:

Reviewer comments:

Reviewer #1

(Remarks to the Author)
The authors have addressed most of my previous questions. Therefore, I recommend its publication without further revision.

Reviewer #2

(Remarks to the Author)
The authors' reply to my comments is satisfactory, and the revised manuscript appears significantly improved. I am, in principle, happy to endorse the publication of this study in Nature Communications.

Reviewer #3

(Remarks to the Author)
The relevant issues have been resolved and no further modifications are required.

Reviewer #4

(Remarks to the Author)
I would like to thank the authors for their detailed and thorough revision. The issues raised in my previous report have been satisfactorily addressed. The new data provided have added strength to the manuscript, and the answers were clear and scientifically valid. I do not have any further comments and would like to support publication of the manuscript as it is now.

Open Access This Peer Review File is licensed under a Creative Commons Attribution 4.0 International License, which permits use, sharing, adaptation, distribution and reproduction in any medium or format, as long as you give appropriate credit to the original author(s) and the source, provide a link to the Creative Commons license, and indicate if changes were made.
In cases where reviewers are anonymous, credit should be given to 'Anonymous Referee' and the source.
The images or other third party material in this Peer Review File are included in the article’s Creative Commons license, unless indicated otherwise in a credit line to the material. If material is not included in the article’s Creative Commons license and your intended use is not permitted by statutory regulation or exceeds the permitted use, you will need to obtain permission directly from the copyright holder.
To view a copy of this license, visit https://creativecommons.org/licenses/by/4.0/

REVIEWER COMMENTS

Reviewer #1 (Remarks to the Author):

The authors present an innovative synthetic-biology–guided strategy to direct gold nanoparticles (AuNPs) onto type IV pili of *Synechocystis* sp. PCC 6803 by fusing a gold-binding peptide to PilA1. This targeted assembly creates a conductive nano-bio interface on the cell envelope, lowers interfacial charge-transfer resistance by about 23%, and increases mediator-free photocurrent on planar ITO by approximately 4-fold, while using substantially less gold than non-targeted approaches. The work addresses a key bottleneck in biophotovoltaics—inefficient extracellular electron transfer (EET)—and offers a conceptually appealing, material-efficient route to engineer bio-electronic interfaces with spatial precision. Despite these advances, several issues should be addressed before publication.

Response: Thank you for your positive and insightful comments.

1. In Figure 4d, the TEM image shows AuNPs distributed along pili outside the cell. The interparticle distances (>50 nm) appear too large to support an effective electrical pathway. Additional evidence that pili-localized AuNPs form a percolating, conductive network interfacing with the electrode would substantially strengthen the mechanistic claims.

Response: Thank you for your comments. We agree that demonstrating a percolating network is crucial. The TEM image in Figure 4d shows the targeted enrichment of AuNPs along the pili protruding from the cell. However, during the actual biofilm formation on the electrode, the pili will be flattened and adhered to the cell surface closely, thus the particles will be densely distributed on the cell surface. To directly assess the interparticle distance in the biohybrid electrode, we performed a scanning electron microscopy (SEM) analysis for the air-dried biofilm surface. This analysis revealed that the smallest interparticle distances on the surface of M6G9Δ*pilT* biofilm appeared to be less than 5 nm (Supplementary Fig. 13). It can be speculated that within the three-dimensional biofilm, where cell surfaces overlap, the effective interparticle distances would be even smaller, thus facilitating effective electron transfer and supporting the formation of a conductive network. The new data and interpretation have been added to the revised manuscript (Page 13, Lines 303-309).

2. For photocurrent measurements, “pre-incubated mixture of *Synechocystis* cells and AuNPs were dropped onto an ITO conductive glass and allowed to air-dry.” However, non-adherent cells may gradually detach, as noted in the biofilm formation assay. Please discuss the “loading” effects.

Response: Thank you for your comments. This is an important point regarding experimental stability. Before photocurrent measurements, the cells were dropped onto the ITO coated glass and allowed to air-dry without any immobilization, thus the adhesion of *Synechocystis* cells on the electrode might be weak. To minimize detachment of cells from the electrode, electrolyte was slowly perfused into the

reactor using a peristaltic pump at 0.4 mL min^{-1}. We acknowledge that some biofilm detachment might occur during the photocurrent measurements, which is one factor limiting the long-term photocurrent stability (see response to point #5 and Supplementary Fig. 24). We have clarified the loading and perfusion protocols in the Methods section (Page 27, Lines 708-710) and discuss the implications for stability in the Results section (Page 16, Lines 378-379).

3. The manuscript attributes photocurrent gains to enhanced cell–electrode charge transfer and increased intercellular interactions after AuNP functionalization. Biofilm formation likely also contribute. Please characterize biofilm structure (e.g., thickness, density, porosity) and discuss how these features affect EIS parameters.

Response: Thank you for your comments. We have performed a detailed characterization of biofilm structure using confocal laser scanning microscopy (CLSM) based on chlorophyll fluorescence. The analysis revealed that AuNPs enrichment induced a thicker and denser biofilm in the central zone of the electrode. Without AuNPs, cells are pushed to the edge of the droplet due to surface tension during the drying process (Supplementary Fig. 21). The AuNPs-enhanced cell-cell interaction prevented this migration to the edge, leading to a uniform cell layer with improved effective interfacial area. This structural change directly explains the observed 52% increase in electrochemically active surface area (ECSA) and the correlated decrease in charge-transfer resistance, as a larger population of cells were effectively wired to the electrode. The detailed descriptions can be found on Page 18, Lines 440-448.

4. For strain M6G9ΔpilT treated with AuNPs, Rct decreases by ~20%, and biofilm formation is only slightly improved (Fig. 7c). Yet the photocurrent density increases by ~4.3-fold (Fig. 5b). Please clarify the primary mechanism(s) underlying this disproportionate photocurrent enhancement.

Response: Thank you for your constructive comments. We appreciate this request for mechanistic clarity. The photocurrent enhancement is not due to a single factor but a synergistic combination of improved interfacial electron transfer and increased effective cell loading. At the interface, conductive pili increased the ECSA by 52% and decreased the charge-transfer resistance by 23%. In the biofilm, AuNPs enrichment doubled the thickness of the electroactive biofilm through enhanced intercellular interaction. Moreover, the AuNPs conductive network facilitated the effective electron transfer inside the dense biofilm. In short, AuNPs enrichment doubled both the number of effectively wiring cells and electron transfer efficiency, ultimately improving the photocurrent density by approximately 4-fold. The relevant discussions are added on Pages 19-20, Lines 480-487.

5. Include long-term chronoamperometry under continuous illumination (hours to days) to assess stability beyond light/dark transients. If performance decays, discuss whether the cause is physiological changes, nanoparticle detachment/aggregation, or photodamage.

Response: Thank you for your suggestions. We have performed the longer-term chronoamperometry

under repeated 8-hour light / 3-hour dark cycles to simulate daily operation. The biohybrid systems maintained its 4-fold photocurrent enhancement over controls for 1 or 2 cycles, when light intensity was set at 400 and 200 μmol photons m^{-2} s^{-1}, respectively (Supplementary Fig. 24). Subsequent photocurrent decay was observed for both hybrid and control systems, and this were attributed to cell bleaching of *Synechocystis* and physiological decline under the electrochemical test conditions (continuous illumination, insufficient gas exchange and mass transfer, and lack of carbon source). This indicates the performance limit in our systems is due to cyanobacterial viability under stress, not AuNPs detachment or aggregation. We discussed these factors and the need to consider optimizing and supporting long-term cell health in future BPV design. Please refer to Pages 15-16 (Lines 371-381 and 400-403).

6. Zeta potential measurements in deionized water do not reflect the ionic strength of BG11. Please report zeta potential in BG11 (or a buffer with matched ionic strength) for physiologically relevant interpretation.

Response: Thank you for your suggestion. In fact, all zeta potential measurements were conducted in BG11 medium to ensure physiological relevance. We have corrected the description in the methods section (Page 25, Lines 630-631).

7. Textual edits and typos
"an cutting-edge" → "a cutting-edge"
"an photocurrent" → "a photocurrent"
"can functioned as" → "can function as"

Response: Thank you, corrected as suggested. Please refer to Lines 32, 35 and 65.

Reviewer #2 (Remarks to the Author):

In the manuscript of Wang et al., the authors address a key challenge in biophotovoltaics: the efficient extraction of electrons from photosynthetic microorganisms. They present a targeted biohybrid strategy using genetically encoded gold-binding peptides to direct the assembly of gold nanoparticles (AuNPs) onto the type IV pili of *Synechocystis* sp. PCC 6803. This creates a defined conductive interface that enhances electron transfer from the cells to external electrodes. Their approach claims a reduction in the interfacial resistance by 23% and achieves a four-fold increase in photocurrent density, while requiring significantly less gold compared to non-targeted methods. The study also claims to identify key methionine residues responsible for AuNP binding and demonstrates the potential for functional transfer of AuNPs between cells, suggesting long-term system stability. Overall, the work offers a generalizable framework for engineering conductive interfaces on living cells for applications in biophotovoltaics and other biohybrid technologies.

The manuscript is clearly written, methodologically sound, and visually supported by high-quality

figures (notably, Figure 1, which is exceptionally well designed and informative).

Response: Thank you for your positive comments.

That said, I have one main point of concern that I believe should be addressed before the manuscript is considered for publication.

Major Comment – Specificity of Enhanced Electron Flow to Photocurrent

While the enhanced photocurrent observed in the M6G9ΔpilT + AuNPs strain compared to M6G9ΔpilT alone is compelling, I find it puzzling that the engineered conductive pili appear to facilitate only photosynthetic electron extraction, with no enhancement in dark current. This outcome is unexpected, particularly given that in *Synechocystis*, the photosynthetic and respiratory electron transport chains share common electron carriers and are known to exchange electrons.

The authors should provide a robust mechanistic explanation or at least a thoughtful discussion on why this selective enhancement occurs.

Without such clarification, the interpretation of the photocurrent enhancement remains incomplete.

Response: Thank you for your comments, which is an excellent and insightful point. We conducted specific dark current measurements of *Synechocystis* in the absence and presence of AuNPs, and found no significant enhancement in dark current upon AuNPs treatment (Supplementary Fig. 20). We propose this selectivity arises from the different pools and kinetics of available electrons during light versus dark periods:

(1) For the continuous dark current (Supplementary Fig. 20), intracellular reductants (e.g., NADPH) are likely depleted by basal respiration over the 4-6 hour drying process, leaving a small reservoir of electrons for dark current.

(2) For the alternating light/dark current measurements, photosynthesis actively generates a large flux of electrons during the light period, creating a substantial pool that can be efficiently harvested by the conductive interface. The small dark current enhancement observed after a light period (Fig. 5b) may stem from residual photosynthetic electrons. Thus, the conductive pili could enhance the extraction of a high-flux photosynthetic electron stream but cannot significantly amplify a signal limited by the electron availability in the dark. The relevant description about dark current can be found on Page 14, Lines 335-341.

Additional Comments

1. Clarification on Benchmarking of BPV Performance in the Introduction

The manuscript states:

“It was estimated that the theoretically maximum photocurrent density of BPV is 340 μA cm^{-2}, a value comparable to that of commercial photovoltaic (PV) technology [7].”

This appears to be a misinterpretation of reference [7] (McCormick et al.). That paper refers to an “achievable” current and power output range, not a theoretical maximum. Furthermore:

• The estimate includes both light and dark current components.

• McCormick et al. reported a range of values (340–2460 μA cm^{-2}) based on lighting conditions, not a

single value.
• The claim of comparability to commercial PVs is inconsistent with McCormick et al.'s statement: "... the gap with recently built solar power stations is substantially larger (ca. 70-fold)."
I encourage the authors to revise this section for accuracy and consistency with the original source.

Response: Thank you for your suggestions. We have revised this statement as "the achievable current densities of BPV range from 340-2460 μA cm^{-2} dependent on light photon flux in different geographical locations" for accuracy with the original reference (Page 2, Lines 35-37).

2. Durability of AuNP-Tagged *Synechocystis*
A key factor for the practical application of this biohybrid system is its long-term stability. I would welcome the inclusion of a longer-term chronoamperometry dataset (e.g., over 10 days), to assess how the photocurrent performance of AuNP-tagged cells evolves over time. While not essential for acceptance, such data would significantly strengthen the manuscript.

Response: Thank you for your suggestions. As detailed in our response to Reviewer #1 (Point 5), we have performed stability tests. The biohybrid systems showed maintained its superior performance for the first 1-2 cycles. The subsequent decay was linked to cyanobacterial photo-bleaching under the non-ideal growth conditions of the electrochemical reactors, highlighting a future challenge in optimization or redesign of BPV structure, rather than a failure of the conductive interface itself. These results and their interpretation can be found on Pages 15-16, Lines 371-381.

3. Inheritance of AuNPs in Regenerating Cells
Following the previous point, I am curious about the behavior of AuNPs during *Synechocystis* cell division. If the cells regenerate, do the nanoparticles remain associated with daughter cells, or is re-functionalization necessary? Some discussion or preliminary observation in this direction would be appreciated, as it could have important implications for system scalability and long-term use.

Response: Thank you for your comments. In the initial manuscript, we have demonstrated the AuNPs deposited on photosynthetic cells could transfer from inactivated cells to the fresh cells without compromising its activity across several generations (Figure 6 and Page 16, Line 383-400). However, as noted in our stability tests, the cyanobacterial cells in our current electrochemical setups do not proliferate due to suboptimal growth conditions (light stress, lack of carbon source, etc, as we mentioned above). Therefore, in situ observation of nanoparticle inheritance during division within the current electrochemical setups was not possible. We believe the primary limitation for long-term operation of the current biohybrid system is cell viability and retention on the electrode, rather than the loss of nanoparticles. Design of advanced BPV structures that support cell growth and cell immobilization is essential for further development. Please refer to Page 16, Line 400-403.

Reviewer #3 (Remarks to the Author):

To address the low photosynthetic electron extraction efficiency in bio-photovoltaics (BPV), this study combined synthetic biology with targeted nano-assembly to conduct the following research: They screened the high-affinity gold-binding peptide M6G9, fused its gene to the PilA1 subunit of Synechococcus phycobacterium type IV pili, and simultaneously knocked out the pili retraction gene to construct a hyper-piliated strain; Leveraging M6G9's specific binding to gold nanoparticles (AuNPs), the study achieved ordered assembly of AuNPs on the pili, forming an extracellular conductive network. Ultimately, this approach increased photocurrent density by 4.3-fold, reduced interfacial electron transfer resistance by 23%, and decreased gold consumption by two orders of magnitude while enabling recyclability and maintaining excellent biocompatibility. After carefully reviewing and analyzing the manuscript, we believe the following improvements are still needed:

1. In the targeting strategy, gold forms Au-S bonds with methionine in M6G9. Compared to non-targeting physical adsorption, this chemical interaction is likely to produce distinct characteristic peaks. Therefore, X-ray photoelectron spectroscopy (XPS) can be employed to analyze the chemical state of Au, allowing for the differentiation of the interfacial formation mechanisms between the two strategies at the chemical level and thereby providing a more comprehensive comparative analysis.

Response: Thank you for your comments. Following your excellent suggestions, we performed XPS analysis on AuNPs to determine the chemical state of Au and S before and after interaction with the M6G9 peptide. The results showed the appearance of new peaks in the spectra of Au 4f and S 2p after binding, indication a shift consistent with the formation of Au-S bond (Supplementary Fig. 2). This provides direct chemical evidence differentiating our targeted, covalent-like attachment from mere physical adsorption, strengthening the mechanistic insights. The detailed description can be found on Pages 7-8, Lines 173-181.

2. The exclusive reliance on "ΔpilT+AuNPs (physical adsorption)" as the non-targeting control limits the scope of comparison. To fully substantiate the superiority of the proposed targeting strategy in terms of interfacial consistency and material utilization, it is advisable to include at least two additional non-targeting control groups. A unified comparison of key parameters—such as Au consumption, photocurrent density, and interfacial homogeneity (quantified by TEM analysis)—would provide a more robust evaluation, highlighting the innovation of this study through a systematic target versus non-target comparison.

Response: Thank you for your suggestions. We agree and have expanded our comparison. Two additional non-targeting controls including wild type strain and an engineered strain displaying a non-binding peptide (A6G9) on its pili (A6G9Δ*pilT*) were included for comparison in photocurrent generation and AuNPs binding capacity. Both controls show low AuNPs binding capacity and no significant photocurrent enhancement (Supplementary Fig. 19), reinforcing that the specific M6G9-AuNPs interaction is essential for the observed performance gains. Please refer to Page 14, Lines 331-335.

3. For the 50-65% recovery rate of AuNPs, optimization methods should be proposed to improve the recovery rate.

Response: Thank you for your comment. We have added a sentence proposing potential optimization strategies, such as using peptidases to induce AuNPs release, followed by chromatographic separation or magnetic separation, to improve recovery in future applications. Please refer to Page 16, Lines 394-395.

4. The current approach to AuNP size optimization is limited, as only three sizes were tested. Expanding the range to include smaller (e.g., 5 nm) and larger (e.g., 80/100 nm) particles would help identify the optimal size interval and determine whether performance peaks at 10 nm or is sustained across a broader range (e.g., 5–10 nm). Furthermore, incorporating molecular docking simulations to study M6G9 interactions with different AuNP sizes, along with quantitative TEM analysis of AuNP assembly density on pili, would provide critical evidence for the "binding pocket matching" hypothesis and substantially improve the mechanistic discussion.

Response: Thank you for your suggestions. We have tested additional AuNPs sizes (5 nm, 8 nm and 80 nm). As shown in Figure 5d, the 10 nm AuNPs still yielded in highest photocurrent improvement, with 8 nm and 20 nm followed closely, while 5 nm, 50 nm and 80 nm provide a more modest approximately 2-fold improvement. We also tried molecular docking to study M6G9 interactions with AuNPs at different sizes, but it failed due to the undefined structure of this short peptide. This suggests an optimal size window around 8-20 nm, possibly related to binding pocket matching, though molecular docking was inconclusive for this short peptide. These new results are present on Page 14, Lines 347-350.

5. A thorough review of the article's writing format is required. For instance, "25 mg ml^{-1}" in line 241 should be revised to "25 mg mL^{-1}", and the same applies to instances like "30 μg ml^{-1}, 50 μg ml^{-1}, 20 μg ml^{-1}" in line 562. Additionally, abbreviations in the text should be clearly defined upon first use, such as supplementing "photosynthetic electron transport chains" with "(PETCs)" in the abstract.

Response: Thank you for your comments. We have meticulously reviewed the manuscript, corrected unit formatting (e.g., mL^{-1}), and ensured all abbreviations are defined at first use (e.g., PETCs in the Abstract, Line 18). Corrections are found throughout (e.g., Lines 256, 604-605 and 661).

Reviewer #4 (Remarks to the Author):

Biophotovoltaic systems are promising and sustainable approaches for directly converting sunlight into electrical energy with the help of living photosynthetic microorganisms. Drawing upon photosynthetic electron extraction, especially from engineered bacterial platforms, has gained more recent interest because of its potential to enhance electron transfer efficiencies that are not achievable in naturally occurring strains. In this work, a targeted nano-bioengineering strategy for

enhanced photosynthetic electron extraction has been reported, whereby *Synechocystis* cells have been engineered to assemble gold nanoparticles onto their type IV pili, yielding a conductive interface that significantly lowers electron-transfer resistance and boosts photocurrent output using minimal amounts of gold. The authors have established a well-structured series of experiments that encompass most of the key components necessary for analyzing photosynthetically derived extracellular electron transfer. Generally, their experimental approach is sound and incorporates multiple complementary aspects. The following are my concerns and suggestions:

Response: Thank you for your positive comments.

The authors demonstrate, using CLSM, that the expression of the eCPX(N)-Spacer construct in *E. coli* results in peripheral green fluorescence, which they use to indicate preferential localization near the cell envelope. While CLSM is an appropriate indication, it cannot be used alone to establish that a GFP-fusion protein is surface-exposed. Complementary surface-accessibility assays must be included to support such a claim. Methods such as protease shaving of intact cells (e.g., treatment with proteinase K or trypsin), non-permeable biotinization, or antibody labeling, combined with flow cytometric analysis, would directly test whether the fluorescent domain is accessible on the outside of the bacterial envelope. Furthermore, immunogold labeling followed by electron microscopy would yield ultrastructural confirmation of surface localization. The inclusion of one or more of these orthogonal approaches would substantially strengthen proof of true surface display.

Response: Thank you for your comments. We have conducted two complementary assays to confirm surface exposure.

(1) Proteinase shaving: fluorescence in *E. coli* cells with surface-displayed eGFP significantly decreased after proteinase shaving for one hour, while cytosolic-expressed eGFP was unaffected (Supplementary Fig. 1a).

(2) Antibody labeling and flow cytometric analysis: only induced *E. coli* cells with surface eGFP expression was successfully labeled by an anti-eGFP antibody modified with far-red fluorescence (Supplementary Fig. 1b). These orthogonal approaches confirm the surface-localized display of our fusion proteins, strengthening this foundational claim. Please refer to Page 6, Lines 133-140 for more details.

The terminology and explanation of zeta potential require revision for scientific accuracy. Line 234: the phrase “a decrease in the zeta potential” is misleading, because a reduction in the magnitude of the (negative) zeta potential would be expected to diminish rather than enhance suspension stability. Similarly, the expression at line 518, “reduced the negative potential,” is scientifically incorrect as written. The manuscript wording at present may confuse readers, as although the numerical value becomes more negative, the magnitude of the zeta potential is actually increasing; it is this higher absolute value that strengthens electrostatic repulsion and improves dispersion. I suggest that both be reworded to clearly distinguish between numerical decreases - i.e., more negative values - and increases in the magnitude of the zeta potential, to avoid misunderstandings and ensure the proper

description of the relationship between surface charge and suspension stability.

Response: Thank you for your suggestions. We appreciate this important correction. We have revised the text to accurately describe changes in the magnitude (absolute value) of the zeta potential. Phrases like "a decrease in the zeta potential" have been changed to "a more negative zeta potential" or "an increase in the magnitude of the zeta potential," clarifying that enhanced suspension stability results from stronger electrostatic repulsion (Lines 248, 280, 579).

In the spot assay, the treatment with AuNPs at 12 h gives fewer colonies compared to the other groups, especially when compared to the "without AuNPs at 12 h" condition. This is not sufficient to support the authors' conclusion that nanoparticle enrichment does not affect growth. I recommend that the authors provide quantitative CFU counts from biological replicates at changing time points, accompanied by appropriate statistical analyses. NP-only control spots on agar should also be included to exclude physical or optical artefacts. Alternatively, an orthogonal viability assay-such as CFU enumeration or live/dead staining at 12 h- would strengthen the interpretation. If the viability gets affected, the authors should report the effect size and discuss whether the decrease reflects a transient growth delay or nanoparticle-induced toxicity, and adjust their conclusions accordingly.

Response: Thank you for your comments. We have performed a more rigorous biocompatibility analysis.
(1) Quantitative CFU counts from 6 biological replicates showed no significant impact of 12-h incubation with AuNPs on the viability and growth of strain M6G9Δ*pilT* (Supplementary Fig. 15 and Supplementary Fig. 16a).
(2) Moreover, we applied fluorescence staining using propidium iodide (PI) to assess cell membrane integrity. PI is a membrane impermeable DNA intercalator that only penetrates damaged membranes and indicates the dead cells. The positive control group was the dead cells that treated with 70% isopropanol. For all M6G9Δ*pilT* cells treated with AuNPs, the PI fluorescence remained low, similar to that of pristine cells (Supplementary Fig. 16b), suggesting that the integrity of cell membranes was not affected by AuNPs enrichment. While long-term liquid culture of *Synechocystis* showed slight growth inhibition (Supplementary Fig. 17), the acute assays confirmed excellent biocompatibility for the duration of our biohybrid assembly and testing. Please refer to Page 13, Lines 311-318 for more details.

In this work, the authors introduce a mediator-free biophotovoltaic system that utilizes targeted AuNP enrichment and controllable nanoparticle assembly to engineer a hybrid conductive layer on the photosynthetic cell envelope. This approach is taken in order to enhance extracellular electron export and offer a potentially universal strategy for intensifying extracellular energy conversion. Discussion: Well elaborated; the authors interpret their results in detail; the advantages of their design, the shortcomings of current approaches, and the way to cope with these are clearly outlined. Comparison with previous literature is appropriate and relevant, enabling the reader to understand the technological advancements that have been made. In particular, the choice of type IV pili as a

biological scaffold to build a living biophotovoltaic interface is well motivated, hence justifying the causality between pilus engineering and improved electron extraction.

Response: Thank you again for your positive comments.

REVIEWERS' COMMENTS

Reviewer #1 (Remarks to the Author):

The authors have addressed most of my previous questions. Therefore, I recommend its publication without further revision.

Response: We are grateful to your insightful comments and suggestions, which help us to improve our manuscript.

Reviewer #2 (Remarks to the Author):

The authors' reply to my comments is satisfactory, and the revised manuscript appears significantly improved. I am, in principle, happy to endorse the publication of this study in Nature Communications.

Response: We are grateful to your insightful comments and suggestions, which help us to improve our manuscript.

Reviewer #3 (Remarks to the Author):

The relevant issues have been resolved and no further modifications are required.

Response: We are grateful to your insightful comments and suggestions, which help us to improve our manuscript.

Reviewer #4 (Remarks to the Author):

I would like to thank the authors for their detailed and thorough revision. The issues raised in my previous report have been satisfactorily addressed. The new data provided have added strength to the manuscript, and the answers were clear and scientifically valid. I do not have any further comments and would like to support publication of the manuscript as it is now.

Response: We are grateful to your insightful comments and suggestions, which help us to improve our manuscript.
